# Development and internal validation of triage-stage prediction models for short-term critical outcomes among adult emergency department patients entering observation or hospital-based care: a retrospective study

**DOI:** 10.3389/fmed.2026.1921692

**Published:** 2026-10-07

**Authors:** Mingwei Wu, Yao Liu, Chengli Zhou, Jinfeng Li

**Affiliations:** 1 Department of Emergency Medicine, The Affiliated Hospital, Southwest Medical University, Luzhou, Sichuan, China; 2 Department of Dermatology, The Affiliated Hospital, Southwest Medical University, Luzhou, Sichuan, China

**Keywords:** emergency department, emergency triage, machine learning, SHAP, short-term critical outcomes, XGBoost

## Abstract

**Background:**

Risk stratification at emergency department triage may help identify patients who require closer assessment or monitoring. However, prediction models that rely only on information available at the triage stage remain less well evaluated. This study aimed to develop and internally validate machine learning models based on triage-stage information for predicting short-term critical outcomes in adult emergency department patients and to interpret a representative explainable model using SHAP analysis.

**Methods:**

This retrospective observational study included 1,842 consecutive adult patients who completed standardized electronic triage and subsequently entered an emergency observation or hospital-based care pathway at a tertiary general hospital between January 2022 and December 2025. The prediction time point and landmark were defined as completion of electronic emergency triage. Only patients who were free of all outcome components at the landmark were included, and candidate predictors were restricted to routinely available information recorded no later than triage completion. The primary outcome was the first critical event occurring during the subsequent 72 h: intensive care unit admission, endotracheal intubation or invasive mechanical ventilation, cardiopulmonary resuscitation, objectively documented escalation involving an implemented resuscitation-level intervention, or in-hospital death. Additional sensitivity analyses restricted the outcome to intensive care unit admission, intubation or invasive ventilation, cardiopulmonary resuscitation, or death and refitted the model without emergency triage level. The cohort was randomly divided into a development cohort and a 30% hold-out cohort. Because the hold-out cohort had already been examined during the original analysis, it was not considered an independent validation set. The primary internal-validation analysis therefore consisted of 10 repetitions of nested cross-validation within the development cohort, using five stratified outer folds for performance estimation and four stratified inner folds for preprocessing and hyperparameter tuning. Logistic regression, random forest, support vector machine, XGBoost, and LightGBM were evaluated using the same resampling framework. The previously examined exploratory hold-out cohort was retained only for secondary exploratory analysis. Model performance was assessed using discrimination, calibration, classification metrics, Brier score, decision-curve analysis, and SHAP-based interpretation. Bootstrap optimism correction was additionally used to assess overfitting.

**Results:**

All 1,842 included patients were event-free at triage completion, and 214 experienced a short-term critical outcome during the subsequent 72-hour period, corresponding to an event rate of 11.6%. The development cohort included 1,289 patients with 150 events, and the previously examined exploratory hold-out cohort included 553 patients with 64 events. In 10 repetitions of nested cross-validation, the mean AUCs were 0.821 ± 0.012 for logistic regression, 0.817 ± 0.015 for random forest, 0.752 ± 0.018 for support vector machine, 0.831 ± 0.013 for XGBoost, and 0.833 ± 0.014 for LightGBM. XGBoost was highest ranked in 42% of repeated outer-validation results, LightGBM in 48%, and logistic regression in 10%. These primary internal-validation results supported broadly comparable performance among XGBoost, LightGBM, and logistic regression rather than consistent superiority of one algorithm. In the secondary exploratory analysis, the representative XGBoost model achieved an AUC of 0.828 in the previously examined exploratory hold-out cohort. At the development-derived threshold of 0.106, its accuracy was 0.770, sensitivity 0.656, specificity 0.785, positive predictive value 0.286, negative predictive value 0.946, F1 score 0.398, and Brier score 0.080. Its paired comparisons, sensitivity analyses, calibration, decision-curve analysis, and SHAP findings in this cohort were interpreted as exploratory.

**Conclusions:**

Repeated nested cross-validation showed broadly comparable discrimination for XGBoost, LightGBM, and logistic regression in predicting post-triage short-term critical outcomes. XGBoost was retained as a representative explainable model, whereas its performance and benchmark comparisons in the previously examined exploratory hold-out cohort should be regarded as secondary exploratory analyses. The study does not establish the superiority or independent validation of XGBoost. Evaluation in a genuinely unused temporal cohort and external prospective validation remain necessary before clinical implementation.

## Introduction

Emergency departments (EDs) must identify patients with potentially serious or time-sensitive conditions under conditions of limited information, variable acuity, and substantial time pressure. Triage is therefore a key component of emergency care, providing an initial framework for prioritizing patients according to clinical urgency and available resources. However, triage assessment remains challenging across healthcare systems. Under-triage may delay recognition of patients who require closer monitoring or urgent reassessment, whereas over-triage may contribute to crowding and unnecessary use of limited emergency resources (1). Because clinical deterioration may occur shortly after ED arrival, risk stratification at the triage stage remains an important area for methodological and clinical investigation.

Traditional triage systems rely on structured acuity categories, presenting complaints, vital signs, and the clinical judgment of triage nurses or physicians. These systems are practical and widely used, but their performance may be affected by inter-rater variability, incomplete early information, and the difficulty of integrating multiple risk signals in real time. Electronic triage approaches that combine routinely collected triage information with outcome-based prediction have therefore been explored as potential adjuncts to conventional triage assessment (2). Machine-learning-based electronic triage has also been reported to provide individualized risk estimates for acute clinical outcomes, suggesting that data-driven methods may complement, rather than replace, clinician judgment in the ED (3).

Machine learning has increasingly been applied to emergency triage and early outcome prediction. Using routinely available triage data, Raita et al. reported that machine learning models could predict critical care and hospitalization outcomes in adult ED patients and showed better discrimination than conventional acuity-based approaches in their study setting (4). Other studies have evaluated triage-stage information for predicting hospital admission, suggesting that demographic characteristics, vital signs, arrival mode, and presenting complaints contain prognostic information at the point of ED entry (5). Similar approaches have also been evaluated in pediatric emergency settings, where machine learning models have been used to support prediction of clinical outcomes during ED triage (6). Taken together, these studies suggest that routinely available triage data may be used to generate individualized risk estimates in addition to categorical urgency assignment.

Despite these advances, several issues remain insufficiently addressed. First, many ED prediction models have focused on hospitalization, admission disposition, or triage-level classification, whereas models targeting short-term critical outcomes may be more directly aligned with early recognition of patients who require urgent reassessment or escalation of monitoring. Such outcomes include intensive care unit admission, invasive ventilation, cardiopulmonary resuscitation, escalation to resuscitation-level care, or early death. Second, some models incorporate laboratory tests, imaging results, or post-triage treatment variables. Although these variables may improve predictive performance, they are not always available at the completion of triage and may introduce information leakage if they occur after the intended prediction time point. Third, interpretability remains particularly important in emergency settings, where clinicians may need to understand whether model predictions are based on clinically plausible triage-stage information. Previous studies using XGBoost or other machine learning approaches with initial triage information have shown promising predictive performance for critical care outcomes or critical interventions in ED patients (7, 8). However, additional studies are still needed to evaluate explainable models that rely strictly on triage-stage information and report discrimination, calibration, decision-curve analysis, and model interpretation within a transparent prediction-model framework.

Tree-based gradient boosting methods, including XGBoost and LightGBM, may be useful for ED risk prediction because they can model nonlinear relationships and interactions among heterogeneous predictors such as age, vital signs, triage acuity, arrival mode, mental status, and comorbidities. Practical machine-learning triage algorithms have been explored for identifying patients who may require critical care in the ED, highlighting the potential value of real-time risk estimation using routinely available data (9). Nevertheless, discrimination alone is not sufficient for evaluating a clinical prediction model. Calibration is also important because predicted probabilities should correspond reasonably to observed event rates, especially when risk estimates may be used to inform clinical reassessment (10). Decision curve analysis provides a method for estimating whether a prediction model offers net benefit across a range of threshold probabilities compared with treating all or no patients as high risk (11).

Interpretability is another important consideration for machine learning models in emergency medicine. Explainable artificial intelligence may help clinicians evaluate whether model behavior is clinically plausible and whether predictions are influenced by recognizable triage-stage variables (12). SHAP analysis is commonly used to describe the contribution of individual predictors to model output and to summarize global feature importance in complex models such as XGBoost. Transparent reporting is also essential. The TRIPOD + AI statement emphasizes clear reporting of model development, validation, performance evaluation, and intended use for prediction models developed using regression or machine learning methods (13). These principles are especially relevant for ED triage models because the prediction time point, candidate predictors, outcome definition, and strategies to minimize information leakage must be explicitly defined.

Therefore, this study aimed to develop and internally validate machine learning–based models using information available by completion of emergency triage to predict short-term critical outcomes occurring during the subsequent 72 h among adult ED patients who were event-free at that prediction time point. Logistic regression, random forest, support vector machine, XGBoost, and LightGBM models were developed and compared. Candidate models were compared using repeated nested cross-validation, and XGBoost was retained as a representative explainable model for detailed assessment of threshold-dependent performance, calibration, Brier score, decision-curve analysis, and SHAP-based interpretation. By focusing on routinely available triage-stage information and clinically relevant short-term critical outcomes, this study sought to evaluate an interpretable prediction model that may help inform adjunctive risk stratification at the time of emergency triage.

## Methods

### Study design

This was a single-center retrospective observational study designed to develop and internally validate machine learning–based prediction models for short-term critical outcomes in adult emergency department patients. A landmark design was applied, with the electronic triage-completion timestamp defined *a priori* as the prediction time point and time zero. This timestamp corresponded to finalization of the standardized triage record by the triage nurse. Only patients who remained free of every component of the composite outcome at the landmark were eligible for the analytical cohort. Candidate predictors were restricted to information recorded no later than triage completion, and outcome follow-up extended from immediately after the landmark through the subsequent 72 h. The development cohort was used for model development and resampling-based internal validation. Because the 30% hold-out cohort had already been examined during the original analysis, it could not be considered an independent validation set. Repeated nested cross-validation within the development cohort was therefore designated as the primary internal-validation approach. The previously examined exploratory hold-out cohort was retained only for secondary exploratory analyses of discrimination, threshold-dependent performance, calibration, clinical utility, sensitivity, and model interpretation. Its results were not used to support confirmatory claims of model superiority or independent validation.

### Setting

This study was conducted in the emergency department of a tertiary general hospital. Consecutive adult patients who presented to the emergency department between January 2022 and December 2025 were retrospectively screened. Emergency triage was performed according to the routine institutional triage process by trained triage nurses. Triage-stage information, including demographic characteristics, mode of arrival, chief complaint, triage acuity, mental status, vital signs, pain score, and selected comorbidities, was recorded as part of routine emergency care. The hospital administrative system recorded 229,245 emergency department encounters during the study period. This hospital-wide census included all administratively registered emergency department encounters and therefore differed from the more restricted population of adult patients who completed standardized electronic emergency triage and subsequently entered the prespecified observation or hospital-based care pathway.

### Participants

Patients were eligible if they were aged 18 years or older, had completed standardized electronic emergency triage, remained free of every component of the composite critical outcome at the triage-completion landmark, and subsequently entered an emergency observation or hospital-based care pathway between January 2022 and December 2025. Cohort identification began with all 229,245 emergency department encounters recorded in the hospital administrative system during the study period. Of these, 61,384 encounters involved adults aged 18 years or older who completed standardized electronic emergency triage. Among these encounters, 2,518 subsequently entered the prespecified emergency observation or hospital-based care pathway, as identified from the recorded emergency department disposition and downstream care-location fields. Records were then deterministically linked using the unique encounter identifier across the electronic triage, emergency observation, procedure, intensive care unit admission, hospital admission, and in-hospital outcome databases. Successful linkage was achieved for 2,247 encounters (89.2%). The remaining 271 encounters could not be linked because of a missing or inconsistent encounter identifier or the absence of a corresponding downstream record. The 2,247 successfully linked encounters constituted the potentially eligible source cohort, including 522 encounters in 2022, 548 in 2023, 567 in 2024, and 610 in 2025. Case identification was not restricted by calendar date, clinical shift, weekday, season, presenting complaint, triage level, or emergency department treatment area. Electronic timestamps for emergency department arrival, triage completion, and each potential outcome event were compared for every candidate encounter. Any patient who experienced an outcome component before or at triage completion was ineligible for the landmark cohort. For patients with repeated emergency department visits, only the first eligible visit was retained to avoid within-patient correlation. We excluded 96 repeat visits, 17 encounters with death documented before or at triage completion, 126 patients with at least one missing required triage-stage predictor, 74 patients transferred to another institution before complete outcome ascertainment, and 92 patients whose complete 72-hour outcome could not be verified. After these exclusions, 1,842 unique adult patients were included in the final analysis, comprising 1,289 patients in the development cohort and 553 patients in the previously examined exploratory hold-out cohort.

### Outcome

The primary outcome was the first short-term critical event occurring after completion of electronic emergency triage and within the subsequent 72 h. The triage-completion timestamp served as the landmark and time zero. A qualifying event was required to occur during the interval greater than 0 to 72 h after the landmark. The composite outcome comprised five prespecified components. Intensive care unit admission was defined as documented physical admission or transfer to an intensive care unit, rather than an ICU consultation or admission request alone. Endotracheal intubation or invasive mechanical ventilation required a time-stamped airway-procedure or mechanical-ventilation record. Cardiopulmonary resuscitation required documented chest compressions or advanced cardiac life-support resuscitation. In-hospital death required a documented death occurring within the 72-hour post-landmark period. Escalation to emergency resuscitation or critical care management required post-landmark activation of resuscitation-level care accompanied by at least one objectively documented intervention: initiation of vasopressor infusion, noninvasive ventilation or high-flow nasal oxygen for acute respiratory failure, emergency electrical cardioversion or defibrillation, or activation of a massive transfusion protocol. Transfer to a resuscitation area alone, an upgraded triage category, a consultation request, a physician order without documented implementation, or closer observation without a qualifying intervention did not satisfy the escalation definition. Events occurring before or at the landmark were not classified as post-landmark outcomes, and patients with any such event were ineligible for the analytical cohort. When more than one component occurred, the time of the first qualifying component defined the outcome time, while all components occurring within 72 h were retained for the component-frequency and overlap analyses. Patients who remained free of all components throughout the post-landmark period were classified as having non-critical outcomes.

Potential outcome events and their timestamps were independently adjudicated by two emergency physicians using emergency department disposition records, resuscitation records, procedure records, intensive care unit admission records, medication-administration records, respiratory-support records, and in-hospital outcome documentation. The adjudicators were blinded to model predictions and to the candidate predictor dataset, including the recorded emergency triage level. Disagreements were resolved through review by a third senior emergency physician.

### Variables

Candidate predictors were restricted to variables documented no later than the electronic triage-completion landmark to ensure that all predictor information preceded the outcome-risk interval. Candidate variables included age, sex, arrival by ambulance, chief complaint category, emergency triage level, altered mental status, systolic blood pressure, heart rate, respiratory rate, body temperature, peripheral oxygen saturation, pain score, cardiovascular disease, respiratory disease, diabetes mellitus, and chronic kidney disease. Age was analyzed as a continuous variable. Sex was categorized as female or male. Arrival by ambulance was coded as yes or no. Chief complaint was grouped into chest pain, dyspnea, abdominal pain, neurologic symptoms, trauma, fever, and other complaints. Emergency triage level was analyzed as an ordinal acuity indicator and was encoded according to the institutional triage scale, where lower numerical levels indicated higher acuity. Altered mental status was coded as present or absent according to triage documentation. Vital signs included systolic blood pressure, heart rate, respiratory rate, body temperature, and SpO₂. Pain score was assessed using the numeric rating scale. Comorbidities were identified from the emergency medical record and previous medical history documented at presentation. For descriptive analyses, clinically meaningful derived variables were also summarized, including age ≥65 years, systolic blood pressure <90 mmHg, heart rate ≥110 beats/min, respiratory rate ≥24 breaths/min, and SpO₂ < 93%. These derived variables were used only for baseline description and were not simultaneously entered into the primary machine learning models together with their corresponding continuous variables, in order to avoid redundant representation of the same clinical information.

### Data sources and data handling

Data were obtained from the electronic emergency medical record system, triage information system, nursing triage records, vital sign monitoring records, emergency physician documentation, and hospitalization outcome records. Demographic characteristics, mode of arrival, chief complaint, triage level, mental status, vital signs, pain score, and comorbidities were extracted from records generated at the time of emergency triage. All predictors were defined using information recorded before or at the electronic triage-completion landmark. Variables recorded after the landmark, including subsequent diagnostic findings, laboratory results, imaging interpretations, therapeutic interventions, disposition decisions, or inpatient-management information, were not used as candidate predictors. Arrival, triage-completion, and outcome timestamps were ordered chronologically to confirm that predictor ascertainment preceded the outcome-risk interval. Continuous variables were checked for range plausibility, and categorical variables were checked for allowable values. Predictor-specific missingness was evaluated among the 2,134 candidate records remaining after the exclusion of repeat visits and patients who were dead on arrival. Age, sex, emergency triage level, and chief complaint category were complete. Missing values were identified for arrival mode in 5 patients (0.23%), altered mental status in 8 (0.37%), systolic blood pressure in 34 (1.59%), heart rate in 29 (1.36%), respiratory rate in 43 (2.01%), body temperature in 55 (2.58%), SpO₂ in 49 (2.30%), pain score in 71 (3.33%), cardiovascular disease in 17 (0.80%), respiratory disease in 15 (0.70%), diabetes mellitus in 12 (0.56%), and chronic kidney disease in 14 (0.66%). Because more than one predictor could be missing for an individual patient, predictor-specific counts were not mutually exclusive. Overall, 126 patients (5.90%) had at least one missing required predictor and were excluded from the complete-case primary analysis. No single predictor had more than 3.33% missingness. Because the overall proportion of patients with any missing required predictor was 5.90%, the primary analysis used the prespecified complete-case cohort. Missing values were not imputed after cohort allocation, and no information from the exploratory hold-out cohort was used to inform preprocessing. The potential selection bias associated with complete-case exclusion was considered when interpreting the findings. Patients with missing key triage predictors or unavailable 72-hour outcome information were excluded from the final analysis. The complete case-identification and exclusion process, including predictor-specific missingness, was documented to permit assessment of potential selection bias.

### Bias and confounding considerations

Several steps were taken to mitigate potential sources of bias in model development and evaluation. First, consecutive eligible patients were identified using prespecified electronic linkage criteria throughout the four-year study period, without restriction by calendar date, clinical shift, weekday, season, presenting complaint, triage level, or emergency department treatment area. Second, for patients with repeated emergency department visits, only the first eligible visit was included to avoid within-patient correlation. Third, an explicit landmark design was applied. Completion of electronic emergency triage was defined as time zero; eligibility required freedom from every outcome component at that time; predictors were restricted to information recorded no later than the landmark; and only events occurring during the subsequent 72-hour period were classified as outcomes. This temporal ordering minimized the risk that the models included information or events occurring after the intended prediction time point. Fourth, each outcome component was operationally defined using time-stamped disposition, procedure, resuscitation, treatment-implementation, or death records. Triage-level upgrading, transfer to a resuscitation area, consultation, or a physician order without implementation was not itself considered an outcome. Outcome adjudication was performed independently of the candidate predictor dataset and model predictions. Because ICU admission and treatment-based outcomes could nevertheless be influenced by clinician decisions and local practice, sensitivity analyses used an objective-only composite and refitted the model without emergency triage level. Fifth, because the original hold-out cohort had already been examined, it was not described as an independent validation set. Primary internal validation was instead based on repeated nested cross-validation within the development cohort, with preprocessing, dummy-variable encoding, and hyperparameter tuning repeated within each inner resampling loop and performance estimated exclusively in the outer folds. The previously examined exploratory hold-out cohort was retained only for secondary exploratory analysis. Sixth, all candidate models were evaluated within the same repeated nested cross-validation framework in the development cohort. No candidate algorithm was designated as superior on the basis of a prespecified hierarchical model-selection rule. XGBoost was retained only as a representative explainable model for detailed secondary analyses, and this choice was not interpreted as evidence of superior predictive performance.

Because this was a retrospective single-center study, some sources of bias could not be fully eliminated. Exclusion of patients with missing key triage information, transfer before outcome ascertainment, or unavailable 72-hour outcome data may have introduced selection bias. Routinely recorded triage variables may also be affected by documentation variability, measurement error, and differences in triage nurse assessment. In addition, this study was designed for prediction rather than causal inference. Candidate variables were selected according to clinical relevance, routine availability at triage, and potential predictive value, rather than to estimate causal effects. Therefore, model outputs, feature importance rankings, and SHAP values should be interpreted as information related to model prediction, not as confounder-adjusted causal effects or independent etiologic risk factors.

### Study size

Among 61,384 adult encounters completing standardized electronic emergency triage, 2,518 subsequently entered the prespecified emergency observation or hospital-based care pathway, and 2,247 were successfully linked across the required clinical databases. After application of the predefined eligibility and complete-case criteria, 1,842 unique adult patients were included, of whom 214 developed the primary outcome. The development cohort included 1,289 patients with 150 outcome events, and the previously examined exploratory hold-out cohort included 553 patients with 64 events. No formal *a priori* sample-size target was imposed because the study included all eligible patients identified through the prespecified record-linkage pathway during the study period. The 16 prespecified predictor domains produced 21 model columns after dummy-variable encoding, corresponding to approximately 7.1 development-cohort events per model column. We recognized that this effective events-per-parameter ratio was relatively low, particularly for flexible machine-learning models. Predictor complexity was therefore restricted *a priori*, univariable screening and data-driven addition of predictors were avoided, and model stability was evaluated using repeated nested cross-validation and bootstrap optimism correction. The available sample size was considered sufficient for exploratory model development and internal evaluation but not for establishing definitive superiority of one modelling algorithm.

### Quantitative variables

Continuous variables were summarized as mean ± standard deviation for descriptive purposes. Clinically relevant thresholds were additionally used for baseline description, including age ≥65 years, systolic blood pressure <90 mmHg, heart rate ≥110 beats/min, respiratory rate ≥24 breaths/min, and SpO₂ < 93%. In the primary prediction models, continuous variables were retained on their original continuous scale to preserve information and reduce information loss from unnecessary categorization. Categorical variables were encoded as binary or dummy variables as appropriate for model development. Emergency triage level was treated as an ordinal acuity variable.

### Statistical methods

Baseline characteristics were summarized for the total cohort, development cohort, and exploratory hold-out cohort, and were also compared according to short-term critical outcome status. Continuous variables were expressed as mean ± standard deviation and compared using Student's t test. Categorical variables were expressed as number and percentage and compared using the chi-square test or Fisher's exact test, as appropriate. These comparisons were descriptive and were not used to infer causal relationships. A two-sided *P* value <0.05 was considered statistically significant for descriptive comparisons. Five prediction models were developed and compared: logistic regression, random forest, support vector machine, XGBoost, and LightGBM. No hierarchical rule for selecting a single best-performing algorithm had been documented before the original analysis. Accordingly, the candidate models were compared primarily using repeated nested cross-validation, and no algorithm was designated as superior solely on the basis of small differences in AUC or threshold-dependent sensitivity. XGBoost was retained as a representative tree-based model for detailed interpretability and secondary performance analyses; this presentation choice was not considered a prespecified model-selection decision. Four clinical benchmark models or scores were additionally evaluated. The first benchmark used emergency triage level alone. The second was a conventional logistic regression model containing emergency triage level and the five triage-stage physiological measurements used in NEWS2: respiratory rate, SpO₂, body temperature, systolic blood pressure, and heart rate. NEWS2 and MEWS were calculated from their standard component scores using information documented no later than triage completion. Supplemental oxygen status was used only for calculation of NEWS2 and was not included as a predictor in the candidate multivariable models. NEWS2 oxygen-saturation scale 1 was used unless hypercapnic respiratory failure requiring scale 2 was documented at triage. These benchmarks were included to determine whether the full model provided incremental discrimination beyond conventional triage acuity and early-warning scores. Logistic regression was included as a conventional statistical benchmark model, whereas the remaining models were included to evaluate the potential added value of nonlinear machine learning approaches. Candidate predictors were retained based on predefined triage-stage availability and clinical relevance rather than univariable statistical significance alone. Categorical predictors were transformed using appropriate dummy-variable encoding. Sensitivity was reported as a clinically relevant threshold-dependent performance measure but was not used as a prespecified tie-breaking rule for selecting a superior algorithm. Outcome-stratified folds were used throughout cross-validation to preserve approximately equal event prevalence across folds. Synthetic minority oversampling, random oversampling, undersampling, and outcome-dependent case weighting were not used, because the primary objective included estimation of calibrated probabilities in the original study population. Class imbalance was addressed through stratified resampling, evaluation of both threshold-independent and threshold-dependent measures, and determination of the classification threshold using development-cohort cross-validated out-of-fold predictions only. In the previously examined exploratory hold-out cohort, AUCs were compared using paired DeLong tests because all models generated predictions for the same patients. XGBoost was compared with each candidate model and each clinical benchmark. For every comparison, the AUC difference, 95% confidence interval, and two-sided *P* value were reported. These comparisons were classified as secondary exploratory analyses and were not interpreted as independent confirmation of model performance or superiority. No claim of superiority was based solely on overlapping or non-overlapping individual AUC confidence intervals. Calibration of the representative XGBoost model was assessed using the calibration intercept and calibration slope. These parameters were estimated by fitting a logistic regression model with the observed binary outcome as the dependent variable and the logit of the predicted probability as the independent variable. Ideal calibration corresponds to an intercept of 0 and a slope of 1. Confidence intervals for the calibration intercept and slope in the previously examined exploratory hold-out cohort were estimated using 1,000 patient-level bootstrap resamples. Decision-curve analysis was performed in the previously examined exploratory hold-out cohort across threshold probabilities of 0.05–0.30. The representative XGBoost model was compared directly with the conventional logistic regression benchmark combining emergency triage level with respiratory rate, peripheral oxygen saturation, body temperature, systolic blood pressure, and heart rate, as well as with the treat-all and treat-none strategies. Net benefit was calculated at each threshold probability. At the development-derived classification threshold of 0.106, the numbers of true-positive, false-positive, true-negative, and false-negative classifications were additionally reported to describe the potential alert burden and missed-outcome rate. Calibration and decision-curve results from the previously examined hold-out cohort were classified as secondary exploratory analyses. Outcome-component frequencies were reported as non-mutually exclusive counts and percentages, and overlap was summarized according to the number of components experienced by each patient. Three additional sensitivity analyses were conducted to evaluate possible incorporation bias. First, the outcome was restricted to ICU admission, endotracheal intubation or invasive mechanical ventilation, cardiopulmonary resuscitation, or in-hospital death, thereby removing the escalation component. Second, the representative XGBoost model was refitted after excluding emergency triage level from the candidate predictors. Third, the objective-only outcome and exclusion of emergency triage level were applied simultaneously. The representative XGBoost model class and the original development/exploratory hold-out allocation were retained in all sensitivity analyses. Each sensitivity model was fitted and its classification threshold was determined using development-cohort cross-validated predictions before secondary exploratory evaluation in the previously examined hold-out cohort. The primary internal-validation analysis consisted of 10 repetitions of nested cross-validation within the development cohort. Each repetition used five stratified outer folds for performance estimation and four stratified inner folds for preprocessing and hyperparameter tuning. All preprocessing, dummy-variable encoding, and hyperparameter selection were repeated within each inner training partition. Mean AUC, between-repetition standard deviation, and the proportion of outer-validation results in which each model was highest ranked were calculated. In addition, the representative XGBoost development procedure was evaluated using 1,000 bootstrap resamples of the development cohort. Apparent and bootstrap-test performance were used to estimate optimism-corrected AUC and calibration slope. All analyses were performed using R version 4.4.1. The principal packages were pROC for ROC estimation and paired DeLong comparisons, rsample and tune for resampling and tuning, ranger for random forest, kernlab for support vector machine, xgboost for XGBoost, lightgbm for LightGBM, rms for calibration assessment, and fastshap for SHAP-based interpretation. The primary data split used random seed 20260115, and the repeated nested cross-validation and bootstrap procedures used fixed reproducible seed sequences derived from this seed. Predictor preprocessing, tuning ranges, selected hyperparameters, package versions, and random seeds are reported in Supplementary Table S1 and Supplementary Methods.

## Results

### Patient identification and cohort selection

The hospital administrative system recorded 229,245 emergency department encounters between January 2022 and December 2025. Among these, 61,384 encounters involved adults aged 18 years or older who completed standardized electronic emergency triage. A total of 2,518 of these encounters subsequently entered the prespecified emergency observation or hospital-based care pathway. Deterministic linkage using the unique encounter identifier successfully linked 2,247 encounters (89.2%) across the electronic triage and downstream clinical databases; 271 encounters could not be linked because of a missing or inconsistent encounter identifier or the absence of a corresponding downstream record. Of the 2,247 successfully linked encounters, 96 repeat visits and 17 encounters with death documented before or at triage completion were excluded. A further 126 patients were excluded because at least one required triage-stage predictor was missing, 74 because they were transferred to another institution before complete outcome ascertainment, and 92 because their complete 72-hour outcome could not be verified. The final analytical cohort therefore included 1,842 unique adult patients. Review of electronic timestamps confirmed that all included patients were free of every component of the composite outcome at the triage-completion landmark. The complete cohort-selection process is shown in Figure 1.

**Figure 1:**
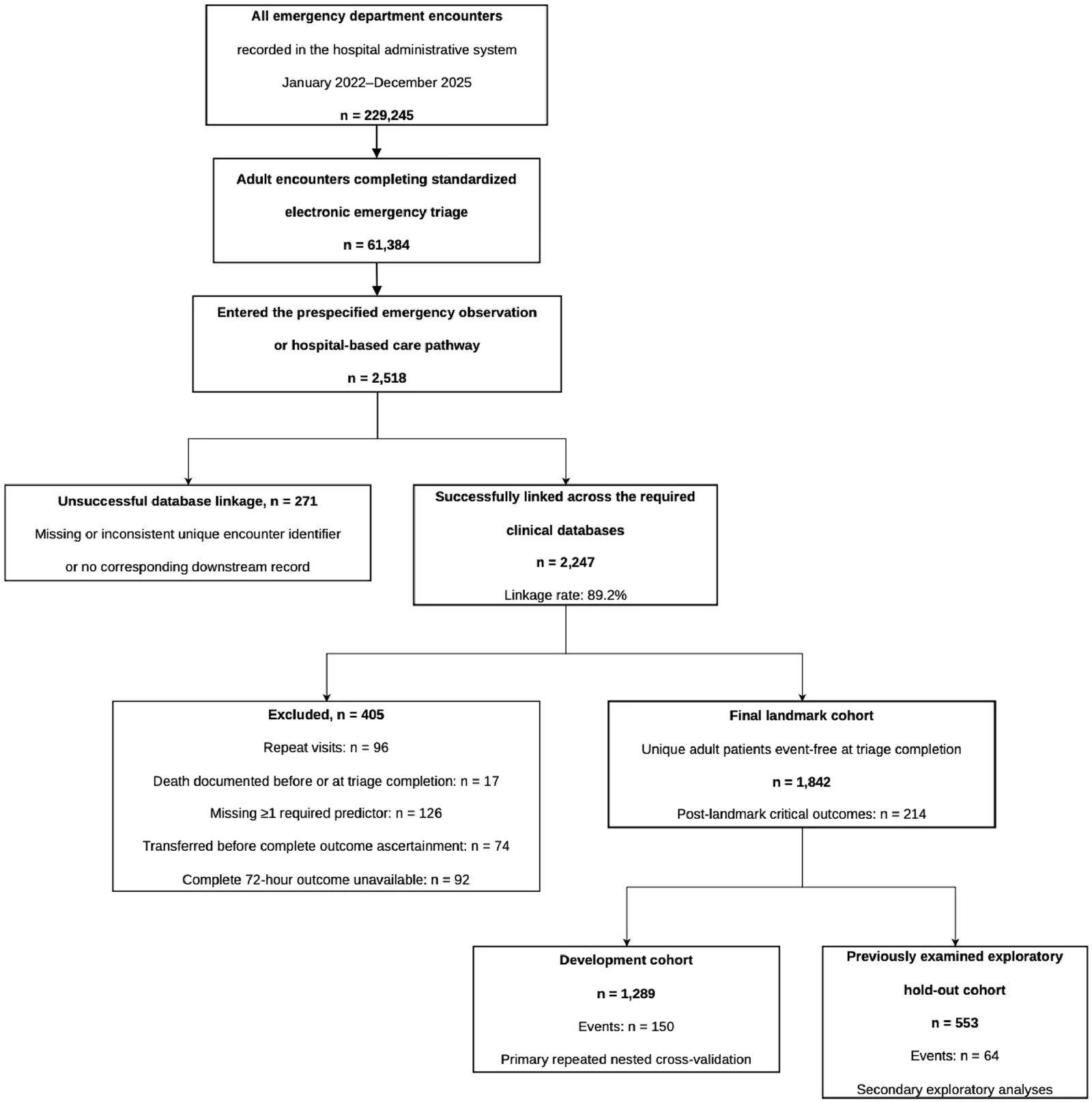
Source-cohort derivation, patient selection, landmark definition, and cohort allocation. The hospital administrative system recorded 229,245 emergency department encounters between January 2022 and December 2025. Of these, 61,384 involved adults who completed standardized electronic emergency triage, and 2,518 subsequently entered the prespecified emergency observation or hospital-based care pathway. Deterministic linkage using the unique encounter identifier successfully linked 2,247 encounters (89.2%) across the required clinical databases; 271 encounters could not be linked because of a missing or inconsistent encounter identifier or the absence of a corresponding downstream record. After excluding 96 repeat visits, 17 encounters with death documented before or at triage completion, 126 patients with at least one missing required predictor, 74 patients transferred before complete outcome ascertainment, and 92 patients without a verifiable 72-hour outcome, the final landmark cohort comprised 1,842 unique adult patients who were event-free at triage completion. Of these, 214 experienced a critical outcome during the subsequent 72-hour period. The final cohort was randomly allocated to a development cohort of 1,289 patients and a 30% hold-out cohort of 553 patients. Repeated nested cross-validation within the development cohort constituted the primary internal-validation analysis. Because the hold-out cohort had previously been examined, it was classified as a previously examined exploratory hold-out cohort and used only for secondary exploratory analyses. ED, emergency department.

### Baseline characteristics of the development and exploratory hold-out cohorts

As shown in Table 1, a total of 1,842 adult emergency department patients were included in this study, including 1,289 patients in the development cohort and 553 patients in the exploratory hold-out cohort. The mean age of the overall cohort was 56.6 ± 18.1 years, and 627 patients (34.0%) were aged ≥65 years. Male patients accounted for 52.2% of the total cohort, and 431 patients (23.4%) arrived by ambulance. The distribution of chief complaint categories and emergency triage levels was generally comparable between the development and exploratory hold-out cohorts. Most patients were assigned to triage level 3 (51.4%), followed by level 4–5 (28.3%), level 2 (15.6%), and level 1 (4.7%). Overall, 77 patients (4.2%) had altered mental status, 57 (3.1%) had systolic blood pressure <90 mmHg, 241 (13.1%) had heart rate ≥110 beats/min, 343 (18.6%) had respiratory rate ≥24 breaths/min, and 352 (19.1%) had SpO₂ < 93%. The overall incidence of short-term critical outcomes was 11.6%, with 150 events in the development cohort and 64 events in the previously examined exploratory hold-out cohort. Most baseline variables were well balanced between the two cohorts, although SBP <90 mmHg and diabetes mellitus differed statistically between groups. These descriptive findings characterize the original data allocation but do not establish the independence of the previously examined exploratory hold-out cohort.

**Table 1:** Baseline characteristics and outcome distribution of the development and exploratory hold-out cohorts.

Variable	Total cohort *n* = 1,842	Development cohort *n* = 1,289	Exploratory hold-out cohort *n* = 553	*P* value
Age, years	56.6 ± 18.1	56.9 ± 17.7	55.7 ± 18.8	0.194
Age ≥65 years	627 (34.0)	451 (35.0)	176 (31.8)	0.208
Male sex	961 (52.2)	671 (52.1)	290 (52.4)	0.920
Arrival by ambulance	431 (23.4)	308 (23.9)	123 (22.2)	0.479
Chief complaint category				0.987
Chest pain	275 (14.9)	192 (14.9)	83 (15.0)	
Dyspnea	233 (12.6)	164 (12.7)	69 (12.5)	
Abdominal pain	237 (12.9)	166 (12.9)	71 (12.8)	
Neurologic symptoms	130 (7.1)	94 (7.3)	36 (6.5)	
Trauma	251 (13.6)	174 (13.5)	77 (13.9)	
Fever	245 (13.3)	175 (13.6)	70 (12.7)	
Other	471 (25.6)	324 (25.1)	147 (26.6)	
Emergency triage level				0.132
Level 1	87 (4.7)	54 (4.2)	33 (6.0)	
Level 2	287 (15.6)	214 (16.6)	73 (13.2)	
Level 3	946 (51.4)	659 (51.1)	287 (51.9)	
Level 4–5	522 (28.3)	362 (28.1)	160 (28.9)	
Altered mental status	77 (4.2)	51 (4.0)	26 (4.7)	0.545
Systolic blood pressure, mmHg	128.7 ± 19.4	128.8 ± 19.1	128.6 ± 20.2	0.827
SBP <90 mmHg	57 (3.1)	31 (2.4)	26 (4.7)	0.014
Heart rate, beats/min	91.1 ± 16.5	90.7 ± 16.3	91.8 ± 17.0	0.228
Heart rate ≥110 beats/min	241 (13.1)	162 (12.6)	79 (14.3)	0.354
Respiratory rate, breaths/min	20.8 ± 3.9	20.8 ± 3.8	20.7 ± 4.0	0.885
RR ≥24 breaths/min	343 (18.6)	243 (18.9)	100 (18.1)	0.747
Body temperature, °C	36.8 ± 0.6	36.8 ± 0.6	36.8 ± 0.6	0.579
SpO₂, %	95.9 ± 3.4	95.9 ± 3.3	96.0 ± 3.5	0.402
SpO₂ < 93%	352 (19.1)	251 (19.5)	101 (18.3)	0.589
Pain score, NRS	3.6 ± 2.0	3.6 ± 2.0	3.7 ± 2.0	0.212
Cardiovascular disease	489 (26.5)	335 (26.0)	154 (27.8)	0.441
Respiratory disease	287 (15.6)	193 (15.0)	94 (17.0)	0.304
Diabetes mellitus	236 (12.8)	148 (11.5)	88 (15.9)	0.011
Chronic kidney disease	97 (5.3)	71 (5.5)	26 (4.7)	0.551
Short-term critical outcome	214 (11.6)	150 (11.6)	64 (11.6)	1.000

Data are presented as mean ± standard deviation or *n* (%). *P* values were calculated using Student's *t* test for continuous variables and chi-square test or Fisher's exact test for categorical variables, as appropriate. SpO₂, peripheral oxygen saturation; SBP, systolic blood pressure; RR, respiratory rate; NRS, numeric rating scale.

### Timing of triage completion and post-landmark outcomes

The median interval from emergency department arrival to completion of electronic triage was 8 min (interquartile range, 5–12 min; range, 2–31 min). None of the 1,842 included patients experienced any component of the composite critical outcome before or at triage completion. The earliest qualifying outcome occurred 16 min after the landmark. Among the 214 patients with a short-term critical outcome, the first qualifying event occurred within 1 h after triage completion in 31 patients (14.5%), at more than 1 to 6 h in 64 (29.9%), at more than 6 to 24 h in 72 (33.6%), and at more than 24 to 72 h in 47 (22.0%).

### Outcome components and overlap

Among the 214 patients with the primary composite outcome, 103 (48.1%) were admitted to an intensive care unit, 58 (27.1%) underwent endotracheal intubation or invasive mechanical ventilation, 22 (10.3%) received cardiopulmonary resuscitation, 96 (44.9%) met the prespecified escalation definition, and 31 (14.5%) died within 72 h after the landmark. Because patients could experience more than one component, these counts were not mutually exclusive. Overall, 141 patients (65.9%) experienced one component, 54 (25.2%) experienced two, 15 (7.0%) experienced three, and 4 (1.9%) experienced four components. The objective-only composite of ICU admission, intubation or invasive ventilation, CPR, or death occurred in 178 patients (9.7% of the full cohort). Sixty patients experienced both escalation and at least one objective component, whereas 36 experienced escalation without an objective component. Component frequencies and overlaps are reported in Table 6.

### Baseline characteristics according to short-term critical outcomes

As shown in Table 2, among the 1,842 patients who were event-free at the triage-completion landmark, 214 developed a short-term critical outcome during the subsequent 72-hour period, whereas 1,628 remained free of the composite outcome. Patients with critical outcomes were significantly older than those without critical outcomes (67.2 ± 16.3 vs. 55.2 ± 17.8 years, *P* < 0.001), and the proportion of patients aged ≥65 years was also markedly higher in the critical outcome group (58.9% vs. 30.8%, *P* < 0.001). Compared with patients without critical outcomes, those with critical outcomes were more likely to be male, arrive by ambulance, have higher-acuity triage levels, and present with altered mental status. Vital signs also differed substantially between groups. Patients with critical outcomes had lower systolic blood pressure and SpO₂ and higher heart rate, respiratory rate, and body temperature than those without critical outcomes. They were also more likely to have SBP <90 mmHg, heart rate ≥110 beats/min, RR ≥24 breaths/min, and SpO₂ < 93% (all *P* < 0.001). In addition, cardiovascular disease, respiratory disease, diabetes mellitus, and chronic kidney disease were more common in the critical outcome group. In contrast, the distribution of chief complaint categories and pain score did not differ significantly between groups. These findings suggest that short-term critical outcomes were strongly associated with older age, higher triage acuity, abnormal vital signs, impaired consciousness, ambulance arrival, and underlying comorbidities.

**Table 2:** Baseline characteristics according to short-term critical outcomes.

Variable	Non-critical outcome *n* = 1,628	Critical outcome *n* = 214	*P* value
Age, years	55.2 ± 17.8	67.2 ± 16.3	<0.001
Age ≥65 years	501 (30.8)	126 (58.9)	<0.001
Male sex	833 (51.2)	128 (59.8)	0.021
Arrival by ambulance	355 (21.8)	76 (35.5)	<0.001
Chief complaint category			0.544
Chest pain	234 (14.4)	41 (19.2)	
Dyspnea	204 (12.5)	29 (13.6)	
Abdominal pain	210 (12.9)	27 (12.6)	
Neurologic symptoms	117 (7.2)	13 (6.1)	
Trauma	220 (13.5)	31 (14.5)	
Fever	221 (13.6)	24 (11.2)	
Other	422 (25.9)	49 (22.9)	
Emergency triage level			<0.001
Level 1	33 (2.0)	54 (25.2)	
Level 2	208 (12.8)	79 (36.9)	
Level 3	872 (53.6)	74 (34.6)	
Level 4–5	515 (31.6)	7 (3.3)	
Altered mental status	56 (3.4)	21 (9.8)	<0.001
Systolic blood pressure, mmHg	130.8 ± 18.4	113.2 ± 20.1	<0.001
SBP <90 mmHg	29 (1.8)	28 (13.1)	<0.001
Heart rate, beats/min	89.3 ± 15.9	104.5 ± 15.3	<0.001
Heart rate ≥110 beats/min	162 (10.0)	79 (36.9)	<0.001
Respiratory rate, breaths/min	20.3 ± 3.6	24.5 ± 4.0	<0.001
RR ≥24 breaths/min	231 (14.2)	112 (52.3)	<0.001
Body temperature, °C	36.8 ± 0.6	37.0 ± 0.5	<0.001
SpO₂, %	96.4 ± 3.0	92.4 ± 3.9	<0.001
SpO₂ < 93%	233 (14.3)	119 (55.6)	<0.001
Pain score, NRS	3.6 ± 2.0	3.6 ± 2.1	0.571
Cardiovascular disease	398 (24.4)	91 (42.5)	<0.001
Respiratory disease	220 (13.5)	67 (31.3)	<0.001
Diabetes mellitus	195 (12.0)	41 (19.2)	0.004
Chronic kidney disease	73 (4.5)	24 (11.2)	<0.001

Data are presented as mean ± standard deviation or *n* (%). *P* values were calculated using Student's *t* test for continuous variables and chi-square test or Fisher's exact test for categorical variables, as appropriate. SpO₂, peripheral oxygen saturation; SBP, systolic blood pressure; RR, respiratory rate; NRS, numeric rating scale.

### Primary repeated nested cross-validation and secondary exploratory hold-out performance

As shown in Table 3A, repeated nested cross-validation was designated as the primary internal-validation analysis. Across 10 repetitions, the mean AUCs were 0.821 ± 0.012 for logistic regression, 0.817 ± 0.015 for random forest, 0.752 ± 0.018 for support vector machine, 0.831 ± 0.013 for XGBoost, and 0.833 ± 0.014 for LightGBM. XGBoost was highest ranked in 42% of the repeated outer-validation results, LightGBM in 48%, and logistic regression in 10%. These findings indicated that the relative ranking of the three best-performing models varied across resamples and did not support consistent superiority of any single algorithm. The 5-fold cross-validated out-of-fold results from the development cohort are presented in Table 3B and Figure 2A. LightGBM and XGBoost achieved AUCs of 0.836 and 0.834, respectively, with identical Brier scores of 0.083. Because the previously described AUC-and-sensitivity hierarchy had not been documented before the original analysis, it was not treated as a prespecified model-selection rule. XGBoost was retained only as a representative explainable model for the detailed threshold-based, calibration, decision-curve, sensitivity, and SHAP analyses. This presentation choice did not imply that XGBoost was superior to LightGBM, logistic regression, or the other candidate models. In the previously examined exploratory hold-out cohort, XGBoost achieved an AUC of 0.828 (95% CI, 0.774–0.882), accuracy of 0.770, sensitivity of 0.656, specificity of 0.785, positive predictive value of 0.286, negative predictive value of 0.946, F1 score of 0.398, and Brier score of 0.080. Performance estimates for all candidate models in this cohort are presented in Table 3B and Figure 2B. Because the hold-out cohort had already been examined during the original analysis, these results were considered secondary and exploratory and were not interpreted as evidence from a genuinely untouched validation set or as confirmation of model superiority.

**Table 3:** Primary repeated nested cross-validation and secondary exploratory hold-out performance.

A. Primary repeated nested cross-validation in the development cohort			
Model	Mean AUC	SD	Highest-ranked outer-validation results
Logistic regression	0.821	0.012	10%
Random forest	0.817	0.015	0%
Support vector machine	0.752	0.018	0%
XGBoost	0.831	0.013	42%
LightGBM	0.833	0.014	48%

B. Development-cohort cross-validated and exploratory hold-out performance									
Model	Evaluation dataset	AUC (95% CI)	Accuracy	Sensitivity	Specificity	PPV	NPV	F1 score	Brier score
Logistic regression	Development CV	0.823 (0.787–0.859)	0.784	0.760	0.787	0.319	0.961	0.450	0.083
Logistic regression	Exploratory hold-out	0.813 (0.759–0.867)	0.756	0.625	0.773	0.265	0.940	0.372	0.080
Random forest	Development CV	0.820 (0.783–0.857)	0.761	0.767	0.760	0.296	0.961	0.428	0.084
Random forest	Exploratory hold-out	0.790 (0.725–0.855)	0.749	0.641	0.763	0.261	0.942	0.371	0.081
Support vector machine	Development CV	0.755 (0.707–0.803)	0.853	0.527	0.896	0.401	0.935	0.455	0.086
Support vector machine	Exploratory hold-out	0.734 (0.650–0.818)	0.850	0.500	0.896	0.386	0.932	0.435	0.083
XGBoost	Development CV	0.834 (0.801–0.866)	0.770	0.747	0.773	0.302	0.959	0.430	0.083
XGBoost	Exploratory hold-out	0.828 (0.774–0.882)	0.770	0.656	0.785	0.286	0.946	0.398	0.080
LightGBM	Development CV	0.836 (0.804–0.868)	0.803	0.700	0.817	0.334	0.954	0.453	0.083
LightGBM	Exploratory hold-out	0.831 (0.779–0.882)	0.801	0.625	0.824	0.317	0.944	0.421	0.080

Panel A presents the primary internal-validation results obtained from 10 repetitions of nested cross-validation within the development cohort. Each repetition used five stratified outer folds for performance estimation and four stratified inner folds for preprocessing and hyperparameter tuning. All preprocessing, dummy-variable encoding, and hyperparameter selection were repeated within each inner resampling loop. “Highest-ranked outer-validation results” indicates the proportion of repeated outer-validation results in which each model achieved the highest AUC. Panel B presents performance based on 5-fold cross-validated out-of-fold predictions in the development cohort and secondary exploratory performance estimates from the previously examined 553-patient hold-out cohort. Because this hold-out cohort had previously been examined, its results were not regarded as independent validation evidence and were not used to establish the superiority of any candidate model. No prespecified hierarchical rule was used to designate a single best-performing algorithm. XGBoost was retained only as a representative explainable model for detailed threshold-dependent, calibration, decision-curve, sensitivity, and SHAP analyses. Classification thresholds were derived from cross-validated out-of-fold predictions in the development cohort and applied unchanged in the exploratory hold-out cohort. AUC, area under the receiver operating characteristic curve; CI, confidence interval; CV, cross-validation; NPV, negative predictive value; PPV, positive predictive value; SD, standard deviation; SHAP, Shapley additive explanations.

**Figure 2:**
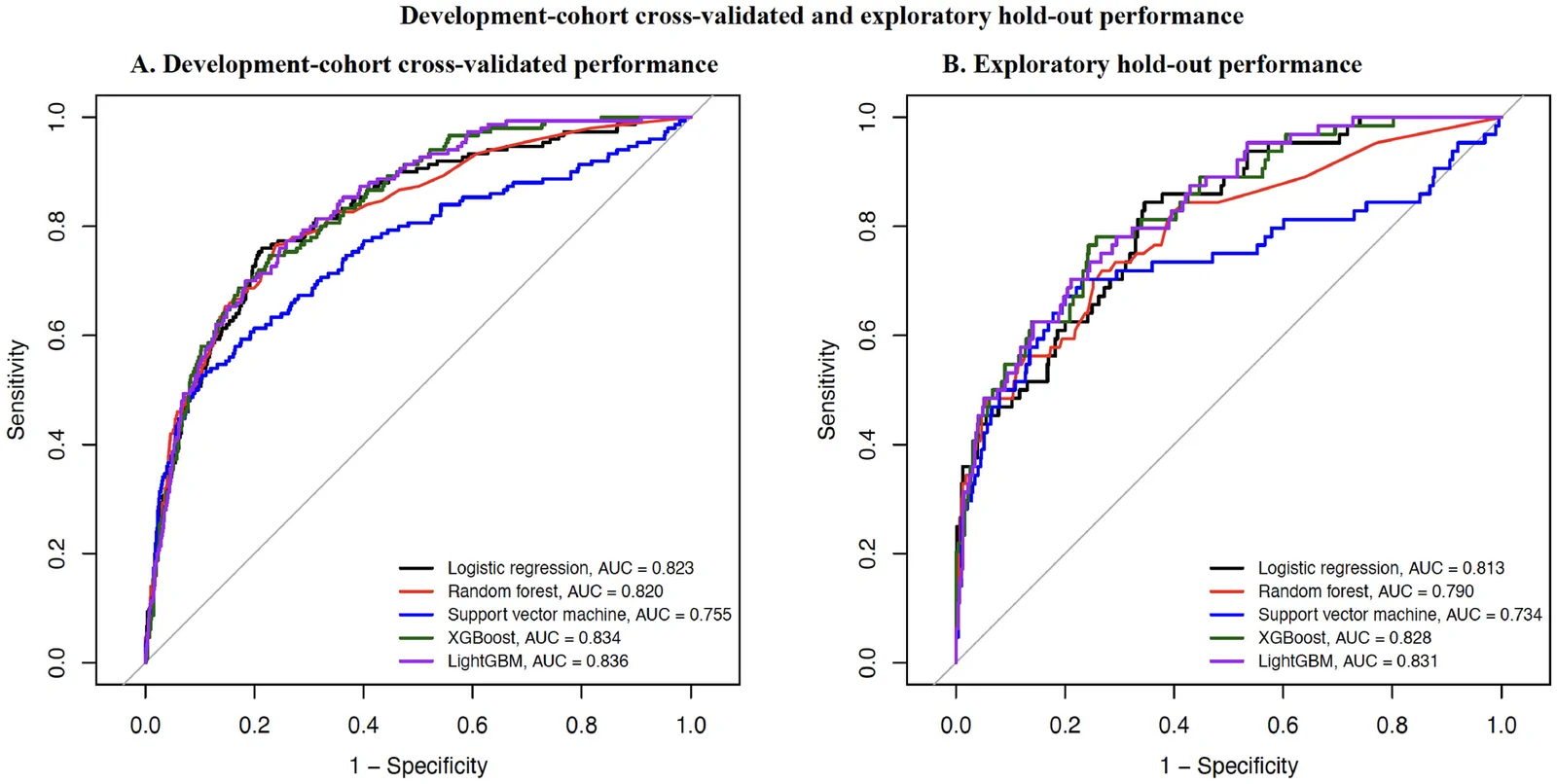
Development-cohort cross–validated and exploratory hold–out performance. **(A)** shows receiver operating characteristic curves derived from 5-fold cross-validated out-of-fold predictions in the development cohort. The AUCs were 0.823 for logistic regression, 0.820 for random forest, 0.755 for support vector machine, 0.834 for XGBoost, and 0.836 for LightGBM. **(B)** shows the corresponding model performance in the previously examined exploratory hold-out cohort, with AUCs of 0.813, 0.790, 0.734, 0.828, and 0.831, respectively. Because the hold-out cohort had already been examined during the original analysis, the results in Panel B are presented as secondary exploratory estimates and are not considered evidence from a genuinely untouched or independent validation set. The primary comparative internal-validation results were obtained using repeated nested cross-validation and are reported in Table 3A. The exploratory hold-out results were not used to establish the superiority of any candidate model. The gray diagonal line represents discrimination equivalent to chance. AUC, area under the receiver operating characteristic curve.

### Exploratory hold-out model and benchmark comparisons

Exploratory paired comparisons in the previously examined exploratory hold-out cohort showed no statistically significant difference between XGBoost and logistic regression (AUC difference, 0.015; 95% CI, −0.010 to 0.040; *P* = 0.238) or between XGBoost and LightGBM (difference, −0.003; 95% CI, −0.021 to 0.015; *P* = 0.744). XGBoost had a higher AUC than random forest (difference, 0.038; 95% CI, 0.008–0.068; *P* = 0.013) and support vector machine (difference, 0.094; 95% CI, 0.047–0.141; *P* < 0.001). These exploratory comparisons suggested that XGBoost, LightGBM, and logistic regression had broadly similar discrimination and did not support general superiority of machine learning over conventional regression. Among the clinical benchmarks, triage level alone had an AUC of 0.746 (95% CI, 0.680–0.812), triage level plus vital signs had an AUC of 0.805 (95% CI, 0.747–0.863), NEWS2 had an AUC of 0.776 (95% CI, 0.710–0.842), and MEWS had an AUC of 0.751 (95% CI, 0.680–0.822). Compared with these benchmarks, the XGBoost AUC was higher by 0.082 (95% CI, 0.041–0.123; *P* < 0.001), 0.023 (95% CI, 0.004–0.042; *P* = 0.018), 0.052 (95% CI, 0.020–0.084; *P* = 0.002), and 0.077 (95% CI, 0.040–0.114; *P* < 0.001), respectively. The relatively small difference between XGBoost and the triage-level-plus-vital-signs model indicates that much of the predictive information was already captured by physiological measurements and triage acuity, although the full model provided a modest incremental improvement. Because these comparisons were conducted in a previously examined hold-out cohort, they were interpreted as secondary exploratory analyses and did not override the primary repeated nested cross-validation findings.

### Primary internal validation and model stability

Across 10 repetitions of nested cross-validation within the development cohort, the mean AUCs were 0.821 ± 0.012 for logistic regression, 0.817 ± 0.015 for random forest, 0.752 ± 0.018 for support vector machine, 0.831 ± 0.013 for XGBoost, and 0.833 ± 0.014 for LightGBM. XGBoost was the highest-ranked model in 42% of repeated outer-validation results, LightGBM in 48%, and logistic regression in 10%. These results indicated that the relative ranking of the three best-performing models was not stable enough to support superiority of one algorithm. In 1,000 bootstrap resamples of the development cohort, the optimism-corrected AUC of the representative XGBoost development procedure was 0.826, compared with the apparent AUC of 0.834. The optimism-corrected calibration slope was 0.94. The small reduction in AUC and calibration slope below 1.0 indicated limited but detectable overfitting. Full stability results and modelling specifications are presented in Supplementary Table S1.

### Exploratory performance, calibration, and clinical utility of the representative explainable XGBoost model

As shown in Table 4, the probability threshold of 0.106 for the representative XGBoost model was derived from development-cohort cross-validated predictions and applied unchanged in the previously examined exploratory hold-out cohort. Among the 553 patients in this cohort, the threshold generated 147 positive alerts, comprising 42 true-positive and 105 false-positive alerts. Of the 64 patients who experienced a short-term critical outcome, 42 were identified and 22 were classified as negative. The corresponding accuracy was 0.770, sensitivity 0.656, specificity 0.785, positive predictive value 0.286, negative predictive value 0.946, F1 score 0.398, and Brier score 0.080. Thus, 71.4% of positive alerts were false positives, and 34.4% of subsequent critical outcomes were missed. The representative XGBoost model had a Brier score of 0.083 in the development cohort and 0.080 in the previously examined exploratory hold-out cohort. In the exploratory hold-out cohort, the calibration intercept was −0.06 (95% CI, −0.51 to 0.40), and the calibration slope was 0.93 (95% CI, 0.69–1.29), with confidence intervals estimated using 1,000 bootstrap resamples. Although the point estimates were close to the ideal values of 0 and 1, respectively, the confidence intervals indicated uncertainty in the calibration parameters. The revised calibration assessment is presented in Figure 3. Exploratory decision-curve analysis directly compared the representative XGBoost model with the triage-level-plus-vital-signs benchmark and the treat-all and treat-none strategies across threshold probabilities of 0.05–0.30. Both prediction models provided positive net benefit over most of the evaluated clinically relevant threshold range. XGBoost showed a modestly greater net benefit than the benchmark across approximately 0.08–0.18. At the development-derived threshold of 0.106, net benefit was 0.053 for XGBoost, 0.047 for the triage-level-plus-vital-signs benchmark, 0.011 for the treat-all strategy, and 0 for the treat-none strategy. These calibration, threshold-dependent, and decision-curve findings were obtained from a previously examined hold-out cohort and were therefore classified as secondary exploratory results rather than independent evidence of calibration or clinical utility. The exploratory decision-curve comparison between XGBoost and the triage-level-plus-vital-signs benchmark is presented in Figure 4.

**Table 4:** Exploratory confusion matrix and diagnostic performance of the representative XGBoost model.

A. Confusion matrix			
Observed outcome	Predicted non-critical	Predicted critical	Total
Non-critical outcome	384	105	489
Critical outcome	22	42	64
Total	406	147	553

B. Diagnostic performance at the development-derived threshold	
Metric	Value
Development-derived probability threshold	0.106
Accuracy	0.770
Sensitivity	0.656
Specificity	0.785
Positive predictive value	0.286
Negative predictive value	0.946
F1 score	0.398
Youden index	0.442
Brier score	0.080

The probability threshold of 0.106 was derived from development-cohort cross-validated predictions and applied unchanged in the previously examined exploratory hold-out cohort. Among 553 patients, the threshold generated 147 positive alerts, comprising 42 true-positive and 105 false-positive alerts. Twenty-two of the 64 patients who subsequently experienced a short-term critical outcome were classified as negative. Thus, false-positive alerts constituted 71.4% of all positive alerts, and 34.4% of subsequent critical outcomes were missed. These results are secondary and exploratory and should not be interpreted as evidence of clinical effectiveness. NPV, negative predictive value; PPV, positive predictive value.

**Figure 3:**
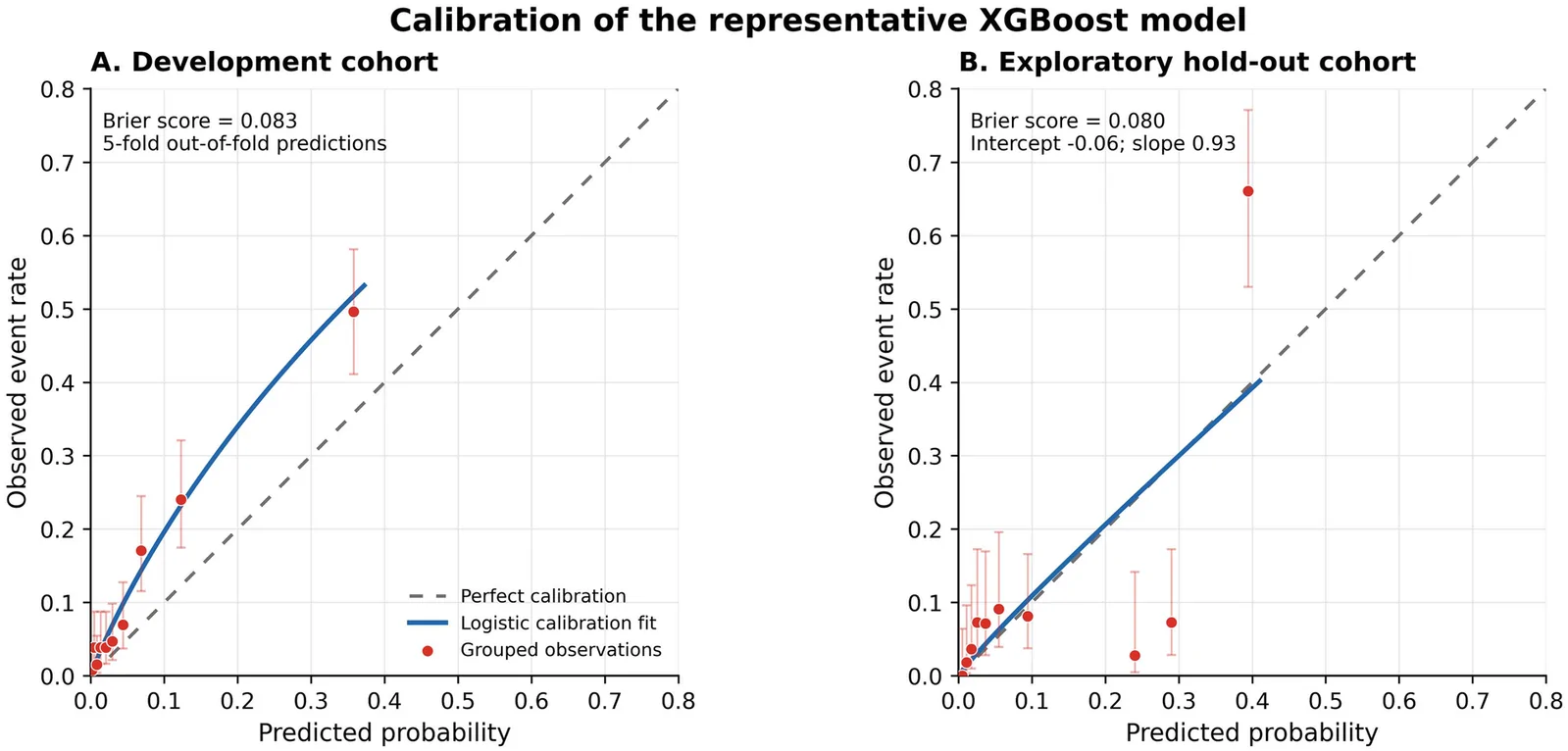
Calibration of the representative XGBoost model in the development and exploratory hold–out cohorts. **(A)** shows calibration based on 5-fold cross-validated out-of-fold predictions in the development cohort. **(B)** shows secondary exploratory calibration in the previously examined hold-out cohort. The solid blue line represents the logistic calibration fit, the dashed gray line represents perfect calibration, and the red points represent observed outcome frequencies across groups of predicted risk, with error bars indicating 95% confidence intervals. The Brier scores were 0.083 in the development cohort and 0.080 in the exploratory hold-out cohort. In the exploratory hold-out cohort, the calibration intercept was −0.06 (95% CI, −0.51 to 0.40), and the calibration slope was 0.93 (95% CI, 0.69–1.29); confidence intervals were estimated using 1,000 bootstrap resamples. Because the hold-out cohort had previously been examined, these findings are classified as secondary exploratory results and require confirmation in an independent temporal or external cohort. CI, confidence interval.

**Figure 4:**
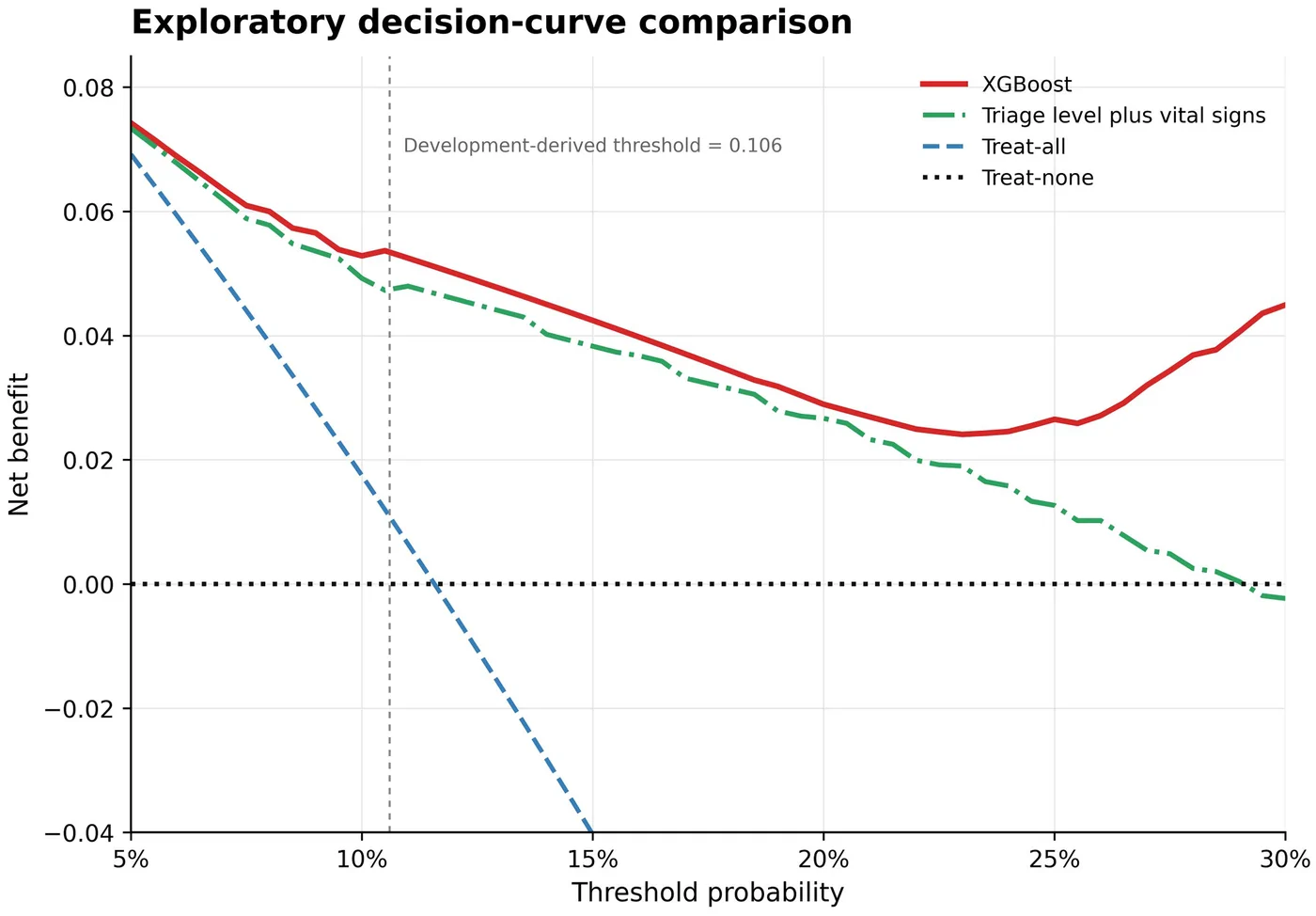
Exploratory decision-curve comparison of the representative XGBoost model and the triage-level-plus-vital-signs benchmark. Decision-curve analysis was performed in the previously examined exploratory hold-out cohort across threshold probabilities of 0.05–0.30. The red solid line represents the representative XGBoost model, the green dash-dotted line represents the conventional logistic regression benchmark combining emergency triage level with respiratory rate, peripheral oxygen saturation, body temperature, systolic blood pressure, and heart rate, the blue dashed line represents the treat-all strategy, and the black dotted line represents the treat-none strategy. Both prediction models provided positive net benefit over most of the evaluated clinically relevant threshold range, although the benchmark approached zero near the upper end of the range. XGBoost showed a modestly greater net benefit than the benchmark across approximately 0.08–0.18. At the development-derived threshold of 0.106, net benefit was 0.053 for XGBoost, 0.047 for the triage-level-plus-vital-signs benchmark, 0.011 for the treat-all strategy, and 0 for the treat-none strategy. Because this analysis was performed in a previously examined hold-out cohort, the findings are secondary and exploratory and do not establish clinical effectiveness.

### Exploratory SHAP-based interpretation of the representative explainable XGBoost model

As shown in Table 5 and Figure 5, SHAP analysis identified SpO₂ as the most important predictor contributing to the representative XGBoost model, with a mean absolute SHAP value of 0.380. Lower SpO₂ values were associated with an increased predicted risk of short-term critical outcomes. Systolic blood pressure was the second most important predictor, and lower systolic blood pressure contributed to higher predicted risk. Emergency triage level, heart rate, age, and respiratory rate were also major contributors to model prediction, with higher-acuity triage levels, higher heart rate, older age, and higher respiratory rate increasing the predicted risk. Other variables, including pain score, ambulance arrival, cardiovascular disease, and respiratory disease, showed smaller but still relevant contributions. Overall, the SHAP results suggested that the representative XGBoost model primarily relied on clinically interpretable triage-stage indicators, particularly oxygen saturation, blood pressure, triage acuity, heart rate, age, and respiratory rate, supporting the clinical plausibility and interpretability of the model.

**Table 5:** Top predictors contributing to the representative XGBoost model in exploratory SHAP analysis.

Rank	Predictor	Mean absolute SHAP value
1	SpO₂	0.380
2	Systolic blood pressure	0.327
3	Emergency triage level	0.278
4	Heart rate	0.253
5	Age	0.239
6	Respiratory rate	0.150
7	Pain score	0.040
8	Arrival by ambulance	0.039
9	Cardiovascular disease	0.029
10	Respiratory disease	0.029

SHAP values were calculated in the previously examined exploratory hold-out cohort to describe the behavior of the representative XGBoost model. The findings are not presented as independent validation evidence. Predictors were ranked according to mean absolute SHAP values. SpO₂, peripheral oxygen saturation; SHAP, Shapley additive explanations.

**Figure 5:**
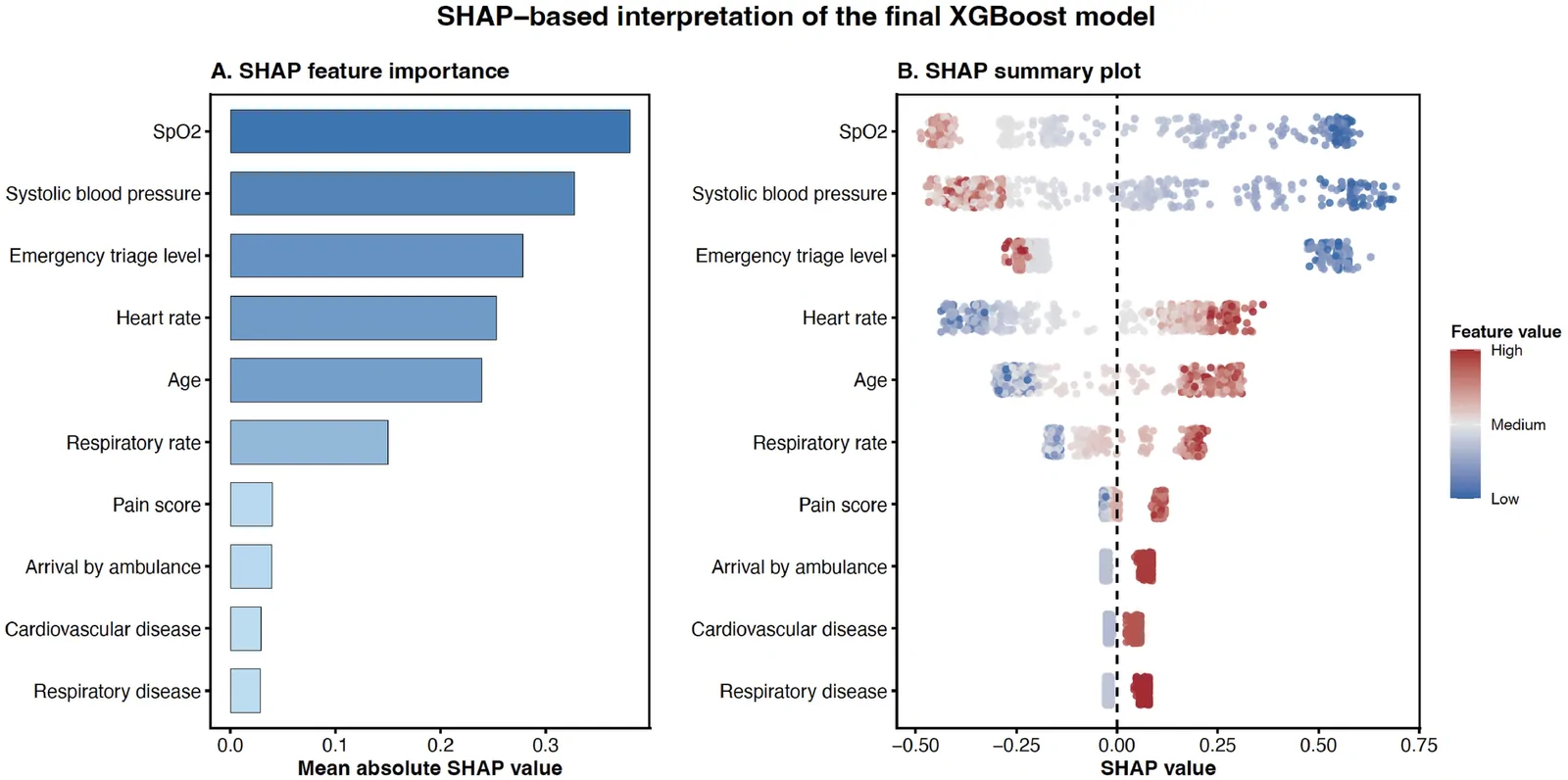
Exploratory SHAP-based interpretation of the representative XGBoost model. SHAP analysis was performed for the representative XGBoost model, and SHAP values were calculated in the previously examined exploratory hold-out cohort. This analysis describes model behavior and is not presented as independent validation evidence. **(A)** shows the 10 predictors with the largest mean absolute SHAP values, representing their overall contributions to model predictions. **(B)** shows the SHAP summary plot; each point represents an individual patient, and color indicates the relative feature value, with red representing higher values and blue representing lower values. Positive SHAP values indicate contributions toward a higher predicted risk of a post-landmark short-term critical outcome, whereas negative values indicate contributions toward a lower predicted risk. For emergency triage level, lower numerical values indicate higher clinical acuity. SpO₂, peripheral oxygen saturation; SHAP, Shapley additive explanations.

### Sensitivity analyses addressing possible incorporation bias

In the secondary exploratory analysis, the representative XGBoost model achieved an AUC of 0.828 (95% CI, 0.774–0.882) in the previously examined hold-out cohort. When the outcome was restricted to ICU admission, endotracheal intubation or invasive mechanical ventilation, cardiopulmonary resuscitation, or death, 178 events remained in the total cohort, including 53 in the exploratory hold-out cohort. Under this objective-only outcome definition, the model achieved an AUC of 0.812 (95% CI, 0.750–0.874), with a sensitivity of 0.642, specificity of 0.792, and Brier score of 0.071 in the exploratory hold-out cohort. When emergency triage level was excluded from the predictors while the primary composite outcome was retained, the corresponding AUC was 0.817 (95% CI, 0.760–0.874), with a sensitivity of 0.641, specificity of 0.779, and Brier score of 0.081. When both restrictions were applied simultaneously, the AUC was 0.801 (95% CI, 0.735–0.867), with a sensitivity of 0.623, specificity of 0.784, and Brier score of 0.072. Although discrimination was moderately attenuated across these analyses, the AUC remained above 0.80 under the most restrictive specification. These findings suggest that neither the escalation component nor emergency triage level alone accounted for the model's discrimination; however, because the analyses were conducted in the previously examined hold-out cohort, they should be interpreted as secondary and exploratory (Table 6). The exploratory hold-out comparisons between XGBoost, the other candidate models, and the clinical benchmarks are summarized in Table 7.

**Table 6:** Outcome-component frequencies, overlap, and sensitivity analyses addressing possible incorporation bias.

A. Outcome components and overlap		
Outcome component or overlap category	Total cohort, *n* (%)	Among primary-outcome events, *n* (%)
Intensive care unit admission	103 (5.6)	103 (48.1)
Endotracheal intubation or invasive mechanical ventilation	58 (3.1)	58 (27.1)
Cardiopulmonary resuscitation	22 (1.2)	22 (10.3)
Escalation involving a qualifying resuscitation-level intervention	96 (5.2)	96 (44.9)
In-hospital death	31 (1.7)	31 (14.5)
Objective-only composite	178 (9.7)	178 (83.2)
Escalation and at least one objective component	60 (3.3)	60 (28.0)
Escalation without an objective component	36 (2.0)	36 (16.8)
One outcome component	141 (7.7)	141 (65.9)
Two outcome components	54 (2.9)	54 (25.2)
Three outcome components	15 (0.8)	15 (7.0)
Four outcome components	4 (0.2)	4 (1.9)

B. Secondary exploratory hold-out sensitivity analyses							
Analysis	Escalation included	Triage level included	Exploratory hold-out events	AUC (95% CI)	Sensitivity	Specificity	Brier score
Primary analysis	Yes	Yes	64	0.828 (0.774–0.882)	0.656	0.785	0.080
Objective-only outcome	No	Yes	53	0.812 (0.750–0.874)	0.642	0.792	0.071
Primary outcome without triage level	Yes	No	64	0.817 (0.760–0.874)	0.641	0.779	0.081
Objective-only outcome without triage level	No	No	53	0.801 (0.735–0.867)	0.623	0.784	0.072

Outcome-component counts are not mutually exclusive because an individual patient could experience more than one component during the 72-hour post-landmark period. The objective-only composite comprised intensive care unit admission, endotracheal intubation or invasive mechanical ventilation, cardiopulmonary resuscitation, or in-hospital death and excluded the escalation component. In each sensitivity analysis, the XGBoost algorithm was retained as the representative explainable model and was refitted using the original development/exploratory hold-out allocation. Model fitting and classification-threshold determination were performed exclusively using development-cohort data before secondary exploratory evaluation in the previously examined hold-out cohort. These additional sensitivity analyses were conducted to assess possible incorporation bias and were not used to establish the superiority of XGBoost or to provide independent validation evidence. AUC, area under the receiver operating characteristic curve; CI, confidence interval; CPR, cardiopulmonary resuscitation; ICU, intensive care unit.

**Table 7:** Exploratory hold-out comparisons of XGBoost with candidate models and clinical benchmarks.

Comparator	Comparator AUC (95% CI)	XGBoost AUC	AUC difference: XGBoost−comparator (95% CI)	*P* value
Logistic regression	0.813 (0.759–0.867)	0.828	0.015 (−0.010 to 0.040)	0.238
Random forest	0.790 (0.725–0.855)	0.828	0.038 (0.008–0.068)	0.013
Support vector machine	0.734 (0.650–0.818)	0.828	0.094 (0.047–0.141)	<0.001
LightGBM	0.831 (0.779–0.882)	0.828	−0.003 (−0.021 to 0.015)	0.744
Emergency triage level alone	0.746 (0.680–0.812)	0.828	0.082 (0.041–0.123)	<0.001
Triage level plus vital signs	0.805 (0.747–0.863)	0.828	0.023 (0.004–0.042)	0.018
NEWS2	0.776 (0.710–0.842)	0.828	0.052 (0.020–0.084)	0.002
MEWS	0.751 (0.680–0.822)	0.828	0.077 (0.040–0.114)	<0.001

AUC differences and 95% confidence intervals were estimated using paired DeLong comparisons in the previously examined 553-patient exploratory hold-out cohort. Because this cohort had already been examined during the original analysis, these comparisons were classified as secondary exploratory analyses and were not used as independent confirmation of model superiority. NEWS2, National Early Warning Score 2; MEWS, Modified Early Warning Score; AUC, area under the receiver operating characteristic curve; CI, confidence interval.

## Discussion

In this single-center retrospective study of 1,842 adult emergency department patients who were event-free at completion of electronic triage, we developed and internally evaluated five prediction models using information available no later than that landmark to predict short-term critical outcomes during the subsequent 72 h. The primary repeated nested cross-validation analysis showed that XGBoost, LightGBM, and logistic regression had broadly comparable discrimination, and their relative rankings varied across resamples. The previously described rule based on a small AUC difference followed by prioritization of sensitivity had not been documented before the original analysis and was therefore not regarded as a prespecified model-selection procedure. Accordingly, the study does not identify a single superior algorithm. XGBoost was retained only as a representative explainable model for detailed secondary analyses, including calibration, threshold-dependent performance, decision-curve analysis, sensitivity analyses, and SHAP-based interpretation. Findings from the previously examined hold-out cohort were interpreted as secondary and exploratory.

The prominent role of vital signs in model prediction is consistent with previous emergency medicine evidence. Abnormal vital signs at triage have been reported to be associated with ICU admission and in-hospital mortality among adult emergency department patients (14). Initial triage vital signs have also been reported to help identify critically ill older adults, consistent with their relevance in early emergency risk assessment (15). In our study, SpO₂, systolic blood pressure, heart rate, and respiratory rate were among the most influential variables in the representative XGBoost model. This pattern is clinically plausible because hypoxemia, hypotension, tachycardia, and tachypnea may serve as early markers of respiratory failure, shock, sepsis, cardiovascular compromise, or other evolving critical illnesses. The importance of these variables is also consistent with studies of early warning scores in emergency department patients, in which physiological derangement has been used to estimate the probability of ICU admission or death (16, 17). Unlike traditional early warning scores, however, the present model integrated multiple triage-stage variables in a nonlinear machine learning framework, potentially allowing more flexible use of age, triage acuity, vital signs, arrival mode, mental status, and comorbidities.

Our findings should be interpreted in the context of previous work on both machine-learning triage models and conventional early-warning systems. Prior studies have shown that machine-learning methods can combine structured triage variables and, in some settings, unstructured chief-complaint information to predict critical care or disposition outcomes (18–21). However, the present paired comparisons did not demonstrate that XGBoost was superior to logistic regression or LightGBM. Repeated nested cross-validation also showed that the relative ranking of these three approaches varied across resamples. Therefore, the present findings support similarity among several modelling approaches rather than general superiority of machine learning. The more relevant incremental comparison was with simpler clinical benchmarks. XGBoost showed higher discrimination than triage level alone, NEWS2, and MEWS, indicating that individualized integration of multiple triage-stage predictors provided information beyond a single acuity category or fixed early-warning score. However, the AUC improvement over a conventional model combining triage level with vital signs was only 0.023. This suggests that much of the model's predictive information arose from established physiological measurements and triage acuity. The incremental contribution of XGBoost was therefore modest and primarily involved flexible nonlinear integration of these variables, additional routinely available clinical information, and SHAP-based individualized interpretation rather than a large gain in discrimination.

An important feature of the present study is the emphasis on interpretability. In emergency departments, a prediction model should not only provide a risk estimate but also allow clinicians to judge whether the model output is based on clinically plausible information. Exploratory SHAP analysis of the representative XGBoost model showed that SpO₂, systolic blood pressure, emergency triage level, heart rate, age, and respiratory rate had the largest mean absolute SHAP values. Because emergency triage level was the third-ranked predictor, we specifically examined whether model performance depended on a variable that might also influence subsequent clinical management. Removal of triage level reduced the exploratory hold-out AUC from 0.828 to 0.817. When the outcome was additionally restricted to ICU admission, endotracheal intubation or invasive mechanical ventilation, cardiopulmonary resuscitation, or death, the exploratory hold-out AUC was 0.801. These findings indicate that triage level contributed useful predictive information but was not solely responsible for the model's discrimination. The continued importance of SpO₂, systolic blood pressure, heart rate, and respiratory rate further suggests that the model captured physiological severity in addition to recorded triage acuity. These variables are familiar to triage nurses and emergency physicians and are routinely assessed at the point of care. This finding is consistent with the clinical plausibility of the model and may reduce concern that the algorithm is primarily influenced by obscure or non-actionable features. SHAP-based interpretation is useful for tree-based models because it can provide both global feature importance and patient-level explanations of model output (22). Nevertheless, SHAP values should be interpreted as model explanations rather than causal effects. As emphasized in discussions of interpretable machine learning for high-stakes clinical decisions, model transparency and clinical plausibility are important, but they do not replace external validation or prospective evaluation before clinical deployment (23).

In the secondary exploratory analysis, the representative XGBoost model achieved an AUC of 0.828 in the previously examined hold-out cohort. This estimate was not statistically different from the corresponding AUCs of logistic regression or LightGBM and should not be interpreted as evidence that XGBoost or machine learning was superior. The relatively low positive predictive value should be considered in the context of the low incidence of short-term critical outcomes. Although the high negative predictive value suggests potential value in identifying patients classified as being at lower risk, the model's moderate sensitivity indicates that some patients who subsequently experience critical outcomes would still be missed. Positive predictions should therefore be regarded as prompts for closer clinical assessment rather than definitive evidence of impending deterioration. Predictions from the representative model should complement, rather than replace, the judgment of triage nurses and emergency physicians, existing triage procedures, and repeated clinical reassessment. These findings are exploratory and require confirmation in an independent temporal or external cohort before any clinical implementation, particularly because patients’ conditions may evolve rapidly after emergency department arrival.

Calibration and potential clinical usefulness are important considerations when evaluating prediction models, because acceptable discrimination does not necessarily imply accurate predicted probabilities or beneficial clinical consequences. In the previously examined exploratory hold-out cohort, the representative XGBoost model had a calibration intercept of −0.06 (95% CI, −0.51 to 0.40) and a calibration slope of 0.93 (95% CI, 0.69–1.29). Although the point estimates were close to the ideal values of 0 and 1, respectively, the confidence intervals were relatively wide and indicated uncertainty in the calibration parameters. These calibration findings therefore require confirmation in an independent temporal or external cohort. Exploratory decision-curve analysis showed that the representative XGBoost model provided a modestly greater net benefit than the triage-level-plus-vital-signs benchmark across part of the clinically relevant threshold range. At the development-derived threshold of 0.106, however, the practical trade-off remained substantial. Applying this threshold to 553 patients generated 147 positive alerts, of which 105 were false positives. Consequently, 71.4% of positive alerts were not followed by a critical outcome, potentially increasing the workload associated with reassessment, monitoring, and alert management. Conversely, 22 of the 64 patients who subsequently experienced a critical outcome were classified as negative, corresponding to a missed-outcome proportion of 34.4%. The threshold therefore cannot be used to rule out subsequent deterioration or replace repeated clinical reassessment. Positive predictions should be regarded as prompts for closer assessment rather than directives for treatment or definitive evidence of impending deterioration. Decision-curve analysis estimates net benefit under specified threshold assumptions and does not demonstrate that model-guided triage improves workflow, resource allocation, or patient outcomes. Furthermore, the observed incremental net benefit over the conventional triage-level-plus-vital-signs benchmark was modest. These findings support further evaluation of the model as a possible adjunct to clinical judgment but do not establish clinical effectiveness (24).

This study has several strengths. First, an explicit landmark design temporally aligned eligibility, predictor ascertainment, and outcome follow-up. Completion of electronic triage served as time zero, all included patients were event-free at that point, predictors were restricted to information recorded no later than the landmark, and the outcome window began immediately thereafter. This design minimized the possibility of temporal leakage from events or clinical information occurring after the intended prediction time point. Second, each component of the primary outcome was operationally defined using time-stamped clinical records, and escalation required implementation of a qualifying resuscitation-level intervention rather than a triage upgrade, transfer decision, consultation, or unimplemented order. Outcome adjudicators were blinded to the predictor dataset and model predictions, and sensitivity analyses using an objective-only outcome and excluding triage level were performed to assess possible incorporation bias. Third, all candidate algorithms were evaluated using a common repeated nested cross-validation framework, which allowed comparative performance and ranking stability to be assessed without relying on a *post hoc* hierarchical selection rule. Repeated nested cross-validation and bootstrap optimism correction were used to evaluate model stability and potential overfitting. XGBoost was retained only as a representative explainable model and was not presented as a superior final algorithm. Fourth, model evaluation included discrimination, calibration, Brier score, decision curve analysis, and SHAP-based interpretation, providing a broader assessment than AUC alone. Finally, the representative model used routinely available clinical information, which may facilitate future evaluation as part of electronic triage or decision-support workflows.

Several limitations should be acknowledged. First, this was a retrospective single-center study without independent temporal or external validation. Although repeated nested cross-validation within the development cohort constituted the primary internal-validation analysis, resampling-based internal validation cannot establish transportability to other hospitals, patient populations, triage systems, or documentation practices. The previously examined hold-out cohort was used only for secondary exploratory analyses and should not be regarded as an independent validation cohort. In addition, the hierarchical rule based on a small AUC difference followed by prioritization of sensitivity had not been documented before the original analysis and was therefore not considered prespecified. XGBoost was retained only as a representative explainable model for detailed secondary analyses, and the present study does not establish that it was superior to LightGBM or logistic regression. The development cohort contained 150 outcome events for 21 encoded model columns, corresponding to approximately 7.1 events per model column. Although predictor complexity was restricted and repeated nested cross-validation and bootstrap optimism correction suggested limited overfitting, this effective ratio remains relatively low for flexible machine-learning models. The absence of statistically significant differences between XGBoost, LightGBM, and logistic regression, together with variation in their rankings across repeated outer-validation results, further precludes identification of a consistently superior algorithm. Independent temporal and external validation are therefore required to assess reproducibility and transportability before any model is considered for broader clinical use (25, 26). Second, the source cohort represented a selected subset of the hospital-wide emergency department population. Although 61,384 adult encounters completed standardized electronic triage during the study period, only 2,518 subsequently entered the prespecified emergency observation or hospital-based care pathway, and 2,247 were successfully linked across the required clinical databases. Patients receiving brief ambulatory management and those discharged without entering this pathway were outside the target source population. In addition, 271 pathway encounters could not be successfully linked, and further exclusions were made for missing predictors, interhospital transfer, and unverifiable outcomes. These restrictions may have introduced selection bias because included patients were likely to have greater illness severity, longer encounters, and more complete electronic documentation than the broader emergency department population. Accordingly, the findings should not be generalized to all emergency department attendees, particularly low-acuity patients discharged after brief ambulatory management. Third, the study relied on routinely recorded triage information, which may be affected by documentation variability, measurement error, and differences in triage nurse assessment. These factors may influence model performance and limit reproducibility in other settings. Fourth, some potentially informative variables, such as laboratory tests, electrocardiographic findings, imaging results, and dynamic reassessment data, were intentionally excluded because they were not consistently available at the completion of triage. This design choice preserved early applicability but may have limited predictive performance. Fifth, although the escalation component required an objectively documented intervention and did not include triage upgrading or transfer to a resuscitation area alone, escalation, ICU admission, and some treatment-based components remain partly dependent on clinician decisions, local protocols, staffing, and resource availability. Incorporation bias therefore cannot be eliminated completely in a retrospective prediction study. However, model discrimination was only moderately reduced when escalation was removed, when triage level was excluded, and when both restrictions were applied simultaneously. These sensitivity findings reduce, but do not eliminate, concern that the observed performance primarily reflected local triage or escalation decisions. Sixth, this study was designed for prediction rather than causal inference. Descriptive differences between patients with and without short-term critical outcomes, as well as SHAP-based feature contributions, should not be interpreted as confounder-adjusted causal effects or independent etiologic risk factors. Residual confounding, unmeasured clinical severity, and unrecorded clinician decision-making may still have influenced both triage-stage variables and subsequent outcomes. Finally, the model's clinical impact was not prospectively tested; therefore, whether use of the model would shorten time to reassessment, reduce delays in escalation, improve patient outcomes, or improve workflow efficiency remains unknown.

Future research should prioritize multicenter external validation, temporal validation, and prospective evaluation studies. In particular, the model should be evaluated in emergency departments with different triage systems, patient volumes, acuity distributions, and resource capacities. Model updating or recalibration may be required before use in new settings. Future studies should also examine how predicted risk can be presented to triage nurses and emergency physicians in a way that is understandable, actionable, and minimally disruptive to clinical workflow. In addition, prospective impact studies are needed to determine whether model-assisted triage can improve clinically meaningful process or patient-centered outcomes, such as time to physician assessment, time to resuscitation, ICU transfer delays, adverse events, and emergency department crowding. Fairness and subgroup performance should also be assessed to ensure that model performance is acceptable across age, sex, disease category, and other clinically relevant subgroups.

In conclusion, among adult emergency department patients entering an observation or hospital-based care pathway, XGBoost, LightGBM, and logistic regression achieved broadly similar discrimination for predicting post-triage short-term critical outcomes. No single algorithm was shown to be consistently superior. XGBoost was retained as a representative explainable model, and its threshold-dependent performance, calibration, benchmark comparisons, clinical-utility assessment, and SHAP findings in the previously examined hold-out cohort should be interpreted as secondary exploratory analyses. Independent temporal or external validation and prospective evaluation are required before clinical use.

## Data Availability

The original contributions presented in the study are included in the article/Supplementary Material, further inquiries can be directed to the corresponding author.
