# Supplementary material for: Development and internal validation of triage-stage prediction models for short-term critical outcomes among adult emergency department patients entering observation or hospital-based care: a retrospective study
Source: Front Med (Lausanne). 2026 Oct 7;13:1921692. doi: 10.3389/fmed.2026.1921692 (PMC13683636; doi:10.3389/fmed.2026.1921692)
Supplement: Supplementary file 1 [file Datasheet1.docx]

**Supplementary Methods**

**Data structure and privacy protection**

Analyses were conducted using a de-identified, one-row-per-encounter analytical dataset. Direct patient identifiers, names, medical-record numbers, contact information, addresses, and other identifying fields were not required for model development and were excluded from the analytical input. The analysis code contains analysis logic only and does not include patient-level data or patient-level predictions. Files generated for reporting contain only aggregate performance estimates, summary tables, or figures.

**Predictor preprocessing**

Candidate predictors were restricted to information documented no later than completion of standardized electronic emergency triage. Continuous predictors were retained on their original continuous scales. Sex and chief-complaint category were represented as categorical variables, and binary predictors were coded as 0 or 1. Emergency triage level was represented as an ordinal numerical variable, with lower values indicating higher acuity.

The primary analysis used the prespecified complete-case cohort. No imputation was performed after allocation of the development and exploratory hold-out cohorts. Dummy-variable encoding was estimated separately within each resampling training partition and then applied to its corresponding assessment partition. For logistic regression and support vector machine models, encoded continuous columns were centered and scaled using means and standard deviations calculated exclusively in the relevant training partition. Random forest, XGBoost, and LightGBM used the encoded but unstandardized predictor matrix.

No preprocessing parameter was estimated from an outer assessment fold or from the exploratory hold-out cohort. Synthetic minority oversampling, random oversampling, undersampling, and outcome-dependent case weighting were not used.

**Cohort allocation and analysis hierarchy**

The complete-case analytical cohort was allocated in an outcome-stratified 70%/30% ratio using random seed 20260115. The development cohort contained 1,289 patients and 150 outcome events. The previously examined exploratory hold-out cohort contained 553 patients and 64 outcome events. Repeated nested cross-validation within the development cohort constituted the primary internal-validation analysis. Analyses performed in the previously examined hold-out cohort were classified as secondary and exploratory and were not interpreted as independent validation evidence.

**Repeated nested cross-validation**

Ten repetitions of nested cross-validation were conducted within the development cohort. Each repetition used five outcome-stratified outer folds for performance estimation. Within each outer-training partition, four-fold stratified inner cross-validation was used for preprocessing and hyperparameter tuning. Dummy-variable encoding, scaling when applicable, and hyperparameter selection were repeated independently within each inner-training partition.

For each candidate algorithm, the hyperparameter specification producing the highest mean inner-fold AUC was selected and refitted using the complete outer-training partition. Predictions were then generated for the corresponding outer-assessment fold. Mean outer-validation AUC, between-result standard deviation, and the proportion of outer-validation results in which each model achieved the highest AUC were calculated.

**Candidate models and hyperparameter tuning**

Five candidate algorithms were evaluated: logistic regression, random forest, support vector machine with a radial kernel, XGBoost, and LightGBM. Logistic regression did not undergo hyperparameter tuning. For the remaining models, candidate configurations were generated within the ranges reported in Supplementary Table S1A. The same nested-resampling structure and outcome-stratified folds were used for all candidate algorithms.

No prespecified hierarchical rule was used to designate one algorithm as superior. XGBoost was retained as a representative explainable model for detailed secondary threshold-dependent, calibration, decision-curve, sensitivity, and SHAP analyses.

**Development-cohort predictions and threshold determination**

Five-fold stratified cross-validated out-of-fold predictions were generated in the development cohort for each candidate model. Classification thresholds were determined using the Youden index calculated exclusively from development-cohort out-of-fold predictions. The resulting thresholds were applied unchanged in the previously examined exploratory hold-out cohort. Accuracy, sensitivity, specificity, positive predictive value, negative predictive value, F1 score, and Brier score were calculated. The development-derived XGBoost threshold was 0.106 and was treated as an illustrative operating point rather than a clinically validated intervention threshold.

**Calibration assessment**

Calibration was evaluated using the Brier score, graphical calibration curves, calibration intercept, and calibration slope. The calibration intercept was estimated using a logistic regression model in which the logit of the predicted probability was included as an offset. The calibration slope was estimated by regressing the observed binary outcome on the logit of the predicted probability. Ideal calibration corresponds to an intercept of 0 and a slope of 1. Confidence intervals for the calibration intercept and slope in the exploratory hold-out cohort were obtained using 1,000 patient-level bootstrap resamples. These estimates were classified as secondary exploratory calibration results.

**Decision-curve analysis**

Decision-curve analysis was conducted in the previously examined exploratory hold-out cohort across threshold probabilities of 0.05–0.30. The representative XGBoost model was compared directly with the conventional logistic regression benchmark combining emergency triage level, respiratory rate, peripheral oxygen saturation, body temperature, systolic blood pressure, and heart rate, as well as with the treat-all and treat-none strategies. At each threshold probability, net benefit was calculated as the proportion of true-positive classifications minus the proportion of false-positive classifications weighted by the odds of the threshold probability. Results were interpreted descriptively because the analysis was performed in a previously examined hold-out cohort.

**Bootstrap optimism correction**

The representative XGBoost development procedure was evaluated using 1,000 patient-level bootstrap resamples of the development cohort. In each bootstrap replicate, the model was fitted in the bootstrap sample and evaluated both in the same bootstrap sample and in the original development cohort. Optimism was calculated as bootstrap-sample performance minus the corresponding original-cohort test performance. Mean optimism was subtracted from apparent development-cohort performance to obtain optimism-corrected AUC and calibration slope. The apparent AUC was 0.834, the optimism-corrected AUC was 0.826, and the optimism-corrected calibration slope was 0.94.

**Outcome overlap**

Outcome components were not mutually exclusive. Intensive care unit admission, endotracheal intubation or invasive mechanical ventilation, cardiopulmonary resuscitation, qualifying escalation, and in-hospital death were counted separately. The number of outcome components experienced by each patient was also calculated. Frequencies and overlap patterns are reported in Table 6A of the main manuscript.

**Sensitivity analyses**

Three sensitivity analyses evaluated possible incorporation bias. First, the outcome was restricted to intensive care unit admission, endotracheal intubation or invasive mechanical ventilation, cardiopulmonary resuscitation, or in-hospital death, excluding the escalation component. Second, emergency triage level was removed from the candidate predictors while the primary outcome was retained. Third, the objective-only outcome and exclusion of emergency triage level were applied simultaneously.

For each analysis, the representative XGBoost model was refitted, and its classification threshold was re-estimated using development-cohort cross-validated predictions. The model and threshold were subsequently evaluated in the unchanged previously examined exploratory hold-out cohort. Complete results are reported in Table 6B of the main manuscript.

**SHAP analysis**

SHAP-based interpretation was performed for the representative XGBoost model. SHAP values were estimated in the previously examined exploratory hold-out cohort using 100 Monte Carlo repetitions per observation. Only aggregate mean absolute SHAP values and graphical summaries were retained for reporting. Patient-level SHAP values were not included in the supplementary or review files.

**Software and reproducibility**

Analyses were performed using R version 4.4.1. Principal packages included pROC for ROC analysis, ranger for random forest, kernlab for support vector machine, xgboost for XGBoost, lightgbm for LightGBM, boot for bootstrap procedures, fastshap for SHAP analysis, and ggplot2 for graphical output. The primary seed was 20260115, and all resampling, bootstrap, and SHAP procedures used deterministic seed sequences derived from this seed. Exact installed package versions were recorded using sessionInfo(). Complete analysis code was uploaded separately through the manuscript submission system for confidential editorial and peer-review purposes; it contains no raw data or patient-identifiable information.

**Supplementary Table S1A. Model tuning ranges and selected hyperparameters**

| **Model** | **Tuning range** | **Selected specification** |
| --- | --- | --- |
| Logistic regression | No hyperparameter tuning | Standard multivariable logistic regression; all prespecified predictors retained |
| Random forest | Number of trees fixed at 1,000; mtry: 3–8; minimum node size: 5–20; sampling fraction: 0.70–1.00 | 1,000 trees; mtry=4; minimum node size=10; sampling fraction=0.80 |
| Support vector machine | Cost: 0.25–8; radial sigma: 0.001–0.05 | Radial kernel; cost=1; sigma=0.01 |
| XGBoost | Trees: 100–500; depth: 2–5; learning rate: 0.01–0.10; minimum child weight: 1–10; subsample: 0.60–1.00; column sample: 0.60–1.00 | 300 trees; depth=3; learning rate=0.03; minimum child weight=5; subsample=0.80; column sample=0.80; gamma=0; L1=0; L2=1 |
| LightGBM | Iterations: 100–500; leaves: 7–31; depth: 3–6; learning rate: 0.01–0.10; minimum observations per leaf: 10–40; feature fraction: 0.60–1.00; bagging fraction: 0.60–1.00 | 300 iterations; 15 leaves; depth=4; learning rate=0.03; minimum observations per leaf=20; feature fraction=0.80; bagging fraction=0.80; L2=1 |

**Supplementary Table S1B. Repeated nested cross-validation stability**

| **Model** | **Mean AUC** | **SD** | **Highest-ranked results** |
| --- | --- | --- | --- |
| Logistic regression | 0.821 | 0.012 | 10% |
| Random forest | 0.817 | 0.015 | 0% |
| Support vector machine | 0.752 | 0.018 | 0% |
| XGBoost | 0.831 | 0.013 | 42% |
| LightGBM | 0.833 | 0.014 | 48% |

**Note:** Results were obtained from 10 repetitions of nested cross-validation within the development cohort. Each repetition used five stratified outer folds for performance estimation and four stratified inner folds for preprocessing and hyperparameter tuning. All dummy-variable encoding, standardization when applicable, and hyperparameter selection were repeated within the inner training partitions. “Highest-ranked results” indicates the proportion of repeated outer-validation results in which each model achieved the highest AUC. AUC, area under the receiver operating characteristic curve; SD, standard deviation.

**Supplementary Table S1C. Reproducibility and resampling specifications**

| **Analysis component** | **Specification** |
| --- | --- |
| Primary data-allocation seed | 20260115 |
| Development/hold-out allocation | Outcome-stratified 70%/30% allocation |
| Primary internal validation | Ten repetitions of nested cross-validation |
| Outer resampling | Five stratified folds for performance estimation |
| Inner resampling | Four stratified folds for preprocessing and hyperparameter tuning |
| Development OOF predictions | Five stratified folds |
| Bootstrap optimism correction | 1,000 patient-level bootstrap resamples of the development cohort |
| Calibration confidence intervals | 1,000 patient-level bootstrap resamples of the exploratory hold-out cohort |
| SHAP Monte Carlo repetitions | 100 per observation |
| Random-number handling | Fixed deterministic seed sequences derived from seed 20260115 |
| Statistical software | R version 4.4.1 |
| Principal packages | pROC, ranger, kernlab, xgboost, lightgbm, boot, fastshap, and ggplot2 |
| Exact package versions | Recorded using sessionInfo() in the aggregate analysis output |
| Code availability | Uploaded separately through the submission system for confidential editorial and peer review |
| Privacy protection | No raw data, direct identifiers, or patient-level predictions included in the review code |
